# Metaheuristic-Optimized Convolutional Neural Network for Automated Diagnosis of Viral Pneumonia and Tuberculosis from Chest X-Rays

**DOI:** 10.3390/diagnostics16101529

**Published:** 2026-05-18

**Authors:** Pamela Hermosilla, Emanuel Vega, Eric Monfroy, Lucas Erazo, Valentina Guzmán, Ricardo Soto

**Affiliations:** 1 Escuela de Ingeniería Informática, Pontificia Universidad Católica de Valparaíso, Valparaíso 2362807, Chile; lucas.erazo@pucv.cl (L.E.); valentina.guzman.e@mail.pucv.cl (V.G.); ricardo.soto@pucv.cl (R.S.); 2 Laboratoire Angevin de Recherche en Ingénierie des Systèmes (LARIS), Université d’Angers, 49000 Angers, France; eric.monfroy@univ-angers.fr

**Keywords:** convolutional neural networks (CNN), metaheuristic optimization, viral pneumonia and tuberculosis, chest X-ray, explainable AI (XAI), hyperparameter tuning

## Abstract

**Background:** Viral Pneumonia and Tuberculosis continue to represent a significant burden on global public health, relying heavily on chest X-rays for screening and diagnosis. Although deep learning systems offer promising diagnostic support, the traditional manual tuning of hyperparameters for Convolutional Neural Networks is often inefficient and computationally expensive, frequently resulting in suboptimal or overly heavy architectures. **Methods:** To address these challenges, this study proposes a hybrid framework that employs metaheuristic algorithms, specifically the Whale Optimization Algorithm, Grey Wolf Optimizer, and Cuckoo Search to automatically optimize the architecture and training parameters of a custom neural network for the multi-class classification of Normal, Viral Pneumonia, and Tuberculosis cases. The proposed approach was evaluated using a rigorous stratified *k*-fold cross-validation protocol on a balanced, multi-source dataset. **Results:** The experimental results demonstrate that the model optimized by the Whale Optimization Algorithm statistically outperforms manually configured baselines, achieving the highest diagnostic accuracy and specificity. Furthermore, a critical finding of this research is the substantial improvement in computational efficiency; the automated optimization reduced the computational load by approximately 74% and the storage requirements by 63%, making the model viable for deployment in resource-constrained environments. **Conclusions:** Finally, to ensure clinical reliability, the decision-making process was validated using Gradient-weighted Class Activation Mapping, which confirmed that the network successfully learns to identify clinically relevant pulmonary structures while ignoring confounding artifacts.

## 1. Introduction

Infectious pulmonary diseases remain a critical global health challenge, imposing a substantial burden on healthcare systems worldwide. Viral Pneumonia is a leading cause of mortality, particularly among children under five and the elderly, accounting for millions of deaths annually [1,2]. Concurrently, Tuberculosis (TB), caused by *Mycobacterium tuberculosis*, persists as one of the most lethal infectious diseases, with latent and active cases requiring rapid identification to prevent spread [3,4].

Chest X-ray (CXR) is the primary and most economically viable diagnostic imaging test for screening these pathologies due to its low cost and wide availability [5,6]. However, manual interpretation of CXRs is fraught with challenges; it is highly dependent on radiologist expertise, prone to inter-observer variability, and susceptible to errors caused by fatigue or high clinical workload [4,7]. Furthermore, the visual patterns of viral pneumonia and TB often overlap with other lung pathologies, complicating accurate differential diagnosis in multi-class scenarios [8,9].

To address these limitations, Computer-Aided Diagnosis (CAD) systems based on Deep Learning (DL), specifically Convolutional Neural Networks (CNNs), have emerged as a powerful solution. Recent literature has demonstrated that CNNs can outperform traditional machine learning methods in feature extraction and classification accuracy for both viral pneumonia [10,11] and tuberculosis [12,13]. Advanced architectures, including ensemble models [1], attention mechanisms [14,15], and hybrid Vision Transformers [4], have further pushed the boundaries of diagnostic performance. However, approximating a realistic clinical scenario requires moving beyond binary tasks to address the more complex 3-class problem: Normal vs. Viral Pneumonia vs. Tuberculosis.

Despite remarkable progress, significant gaps hinder the widespread clinical adoption of these models. First, the performance of DL models is heavily reliant on the optimal configuration of hyperparameters (e.g., learning rate, batch size, filters). Traditional tuning methods, such as grid or random search, are computationally inefficient. Furthermore, standard gradient based optimizers struggle with the highly non convex and rugged loss landscapes of deep architectures, frequently converging to suboptimal local minima [16,17]. Recent evidence in medical imaging suggests that metaheuristic algorithms provide a superior alternative. By treating Hyperparameter Optimization (HPO) as a global optimization problem, they allow for a more exhaustive exploration of the parameter space without the constraints of gradient information [16]. This challenge is fundamentally rooted in the ’No Free Lunch’ (NFL) theorems [18], which establish that no single optimization algorithm can outperform all others across every possible problem. Consequently, the complex and non-linear landscape of medical image features in viral pneumonia and TB necessitates a rigorous search for an optimizer whose search strategy, whether based on swarm intelligence or evolutionary mechanisms, is uniquely aligned with the objective function of clinical diagnostic accuracy. While recent studies have explored stochastic optimization such as Genetic Algorithms for viral pneumonia [19] or Particle Swarm Optimization for TB [13], a systematic comparison of swarm based metaheuristics for tuning custom CNNs in a multidisease context is lacking. Secondly, a challenge persists in balancing computational complexity and efficiency. Many state-of-the-art models rely on heavy, pretrained architectures like DenseNet 121, ResNet 50, or VGG 19 [14,20,21]. While accurate, these models entail high computational costs, limiting their deployment in resource-constrained settings. Conversely, lightweight custom models have shown promise but often require rigorous tuning to match the accuracy of transfer learning approaches [10,12].

Finally, issues related to interpretability and class imbalance create a trust deficit due to the “black box” nature of DL models. Although techniques like Grad-CAM have been applied to visualize pathological regions [4,6], interpretability is often secondary to accuracy. Moreover, there is a lack of studies applying visual interpretability to unified, multi-class optimization protocols rather than simple binary tasks [8,9].

To bridge these gaps, this study proposes a Hybrid CNN-Metaheuristic framework optimized for the multi-class classification of Viral Pneumonia and Tuberculosis. We introduce a custom, lightweight CNN architecture whose hyperparameters are automatically tuned using three distinct global search metaheuristics: Whale Optimization Algorithm (WOA), Grey Wolf Optimizer (GWO), and Cuckoo Search (CS). The selection of these specific metaheuristics is driven by their distinct mechanisms for balancing exploration and exploitation: WOA’s bubble-net hunting mimics local search refinement, GWO provides a hierarchical approach to social leadership in search, and CS utilizes Lévy flights to effectively bypass local optima in high-dimensional CNN parameter spaces, a critical capability given the subtle radiographic differences between viral pneumonia and tuberculosis [22,23]. This approach aims to maximize sensitivity a critical metric for screening infectious diseases while ensuring model efficiency.

This study focuses on the technical evaluation and optimization of the proposed hybrid model. We compare the baseline CNN against its metaheuristic-optimized variants and analyze the trade-offs between computational efficiency and diagnostic performance. Furthermore, following the trend of recent comprehensive surveys [24], our lightweight framework is benchmarked not only against a standard baseline CNN but also against established transfer learning architectures (e.g., ResNet and DenseNet) to demonstrate that metaheuristic-tuned custom models can achieve competitive accuracy with significantly lower computational overhead. While visual interpretability is provided to support diagnostic decision-making, prospective clinical validation is outside the scope of this work.

The main contributions of this work are:

1. Hybrid Optimization Scheme and Sensitivity Analysis: We implement and compare WOA, GWO, and CS to efficiently explore the hyperparameter space of a lightweight CNN. Beyond optimization, we conduct a sensitivity analysis to evaluate how variations in metaheuristic control parameters and batch normalization influence model robustness, providing a deeper understanding of the HPO process in medical contexts.
2. Multi-Class Evaluation: We curate a comprehensive dataset, *ChestX6*, and establish a focused experimental subset to evaluate the model on the challenging 3-class task (Normal/Pneumonia-viral/TB), reporting clinically relevant metrics including Sensitivity, Specificity, F1-score, and AUC.
3. Explainable AI (XAI) Integration: We integrate Grad-CAM++ to generate attribution maps, highlighting radiographic regions driving the model’s predictions to enhance clinical traceability.
4. Reproducible Protocol: We establish a unified preprocessing pipeline and a stratified *k*-fold cross-validation protocol to ensure robust performance estimates.

The remainder of this paper is structured as follows. First, Section 2 reviews the related work on deep learning approaches for lung disease classification, including CNN-based models, advanced architectures, and metaheuristic optimization techniques. Section 3 presents the theoretical background, covering convolutional neural networks, metaheuristic algorithms, explainable AI methods, and validation strategies. Section 4 describes the proposed methodology, including the hybrid CNN–metaheuristic framework, dataset, and evaluation metrics. Section 5 presents the experimental setup and results, followed by a discussion of the findings in Section 6. Finally, Section 7 concludes the paper and outlines future research directions.

## 2. Related Work

The application of Deep Learning (DL) in medical imaging has revolutionized the diagnosis of thoracic diseases. This section reviews the evolution of these techniques, ranging from standard Convolutional Neural Networks (CNNs) and computationally intensive ensemble models to the emerging trend of bio-inspired optimization and explainable artificial intelligence (XAI).

### 2.1. Deep Learning in Lung Disease Classification

Initial efforts in automating lung disease diagnosis successfully established CNNs as a primary tool [1,15]. The success of CNNs in this domain stems from their ability to automatically learn deep, hierarchical, and subtle features from raw image data, a capability particularly valuable for interpreting the complex visual patterns of pulmonary diseases in Chest X-Rays (CXRs) [17].

In the context of viral pneumonia, numerous studies have demonstrated the efficacy of both custom-built CNNs and transfer learning approaches. For instance, Srikeerthi et al. [5] and Katreddi et al. [20] developed architectures to address specific challenges, such as pediatric pneumonia, where anatomical variability complicates diagnosis [25]. While single models like DenseNet-169 have shown effectiveness [20], the current state-of-the-art has shifted towards Ensemble Learning. Yanar et al. introduced PELM, a framework combining InceptionV3, VGG16, ResNet50, and Vision Transformers, achieving 96% accuracy [10]. However, these ensemble approaches, despite their high performance, incur a significant computational cost, requiring the simultaneous training and inference of multiple heavy backbones.

Regarding tuberculosis (TB), the literature reveals a dichotomy between direct classification and segmentation-guided approaches. Classic models like TX-CNN established strong baselines by designing filters specifically for TB textures [26]. More recently, hybrid segmentation-classification strategies have gained prominence. Iqbal et al. proposed a two-stage method (TB-UNet for segmentation followed by DenseNet for classification), achieving nearly 99% accuracy by isolating the lung region [27]. Similarly, the TSSG-CNN model employs semantic segmentation to guide the classifier [28]. Although highly accurate, these methods rely on expensive pixel-level annotations (masks) and multi-stage pipelines that complicate clinical deployment compared to end-to-end classification models.

### 2.2. Advanced Architectures and Optimization Strategies

To overcome the limitations of standard CNNs without resorting to massive ensembles, recent research has focused on integrating Attention Mechanisms and Feature Fusion. It has been demonstrated that incorporating Self-Attention and Squeeze-and-Excitation blocks significantly improves multi-class detection (Normal, Viral Pneumonia, TB, etc.) by recalibrating channel importance [8]. Furthermore, hybrid CNN-Transformer architectures, such as DenseNet combined with ViT, have been proposed to capture both local features and long-range dependencies [15]. Feature fusion has also proven effective; for instance, ResNet-50 backbones have been enhanced by fusing deep features with handcrafted texture descriptors (e.g., LBP, Curvelet transform) to address limitations in feature diversity [6]. Similarly, saliency maps have been utilized as a secondary input stream to compel networks to focus on relevant regions [29]. While these “architectural engineering” approaches improve performance, they often necessitate the manual design of complex modules and extensive trial-and-error to identify optimal configurations.

Beyond architectural design, the performance of CNNs is highly sensitive to their hyperparameter configuration, such as learning rate, batch size, and dropout [30,31]. However, the search space for these parameters is non-convex and high-dimensional, making manual tuning or grid search computationally prohibitive and prone to suboptimal local minima [21]. While approaches like Bayesian optimization tools [14] or stochastic learning strategies [17] have been employed, Metaheuristic Algorithms have emerged as a superior alternative for navigating these complex search spaces. Recent evidence strongly supports the integration of bio-inspired algorithms with Deep Learning. For instance, Genetic Algorithms (GA) applied to VGG-16 optimization have been shown to outperform both manual tuning and custom CNNs in viral pneumonia detection, achieving 98.28% accuracy [19]. Regarding Swarm Intelligence, Particle Swarm Optimization (PSO) has been applied to dynamically update CNN architectures for TB, demonstrating faster convergence and higher accuracy compared to random search [13]. Additionally, the Whale Optimization Algorithm (WOA) has been utilized to tune Fuzzy Neural Networks, further highlighting the efficacy of nature-inspired metaheuristics in medical diagnostics [32].

Furthermore, the efficacy of metaheuristic algorithms in the medical domain extends beyond hyperparameter tuning for supervised deep learning models, proving highly valuable in unsupervised diagnostic paradigms. Traditional clustering algorithms, such as K-Means, are frequently employed for medical data analysis; however, they are notoriously sensitive to initial centroid placement and prone to converging at suboptimal local minima. To address these limitations, recent literature has explored the hybridization of K-Means with advanced metaheuristic algorithms. These hybrid approaches leverage the global search capabilities of metaheuristics to optimize initial cluster configurations, thereby significantly enhancing the clustering accuracy, robustness, and pattern recognition capabilities required for complex medical datasets [33]. Such optimization-based diagnostic studies underscore the versatility of bio-inspired algorithms in overcoming the inherent algorithmic constraints of standard machine learning techniques in healthcare applications, reflecting broader recent trends in applied engineering [34,35].

### 2.3. Explainability (XAI) and Grad-CAM++

Clinical adoption of AI models demands transparency, as “black box” models are unacceptable in healthcare due to their potential reliance on confounding factors such as bones or medical devices rather than actual pathology. Consequently, Grad-CAM and its extension Grad-CAM++ have established themselves as standard mechanisms for validating model focus [36]. Recent studies have made extensive use of these tools to ensure that architectures, including Vision Transformers, direct their attention to lung infiltrates during TB detection [15,36]. Furthermore, these techniques have been utilized to map specific TB manifestations, such as cavities and effusions, to distinct lung zones [4]. Research has also demonstrated that even lightweight models like MobileNetV3 and ShuffleNetV2 can produce coherent activation maps, identifying pathological regions with high fidelity [12]. Beyond confirming true positives, XAI has proven essential for analyzing false positives, revealing instances where models incorrectly focus on anatomical structures like clavicles or the heart shadow [6]. Collectively, these works confirm that XAI is not optional but a requirement for validating the medical relevance of the features learned by the network.

### 2.4. Statistical Validation Protocol: k-Fold Cross-Validation

To ensure the reliability of the results and avoid overfitting to a specific data split, robust statistical validation is essential. The *k*-fold cross-validation method is considered the gold standard in recent high-impact literature. For instance, a 5-fold protocol has been utilized to demonstrate the stability of stochastic learning models, reporting specific metrics for the best-performing fold (99.40%) and analyzing variance across folds [17]. Similarly, 10-fold cross-validation has been employed to provide confidence intervals for models such as Inception-V3 [1]. This rigorous approach contrasts with older studies that relied solely on simple train–test splits, providing a more realistic estimate of the model’s generalization ability in clinical settings.

### 2.5. Summary and Research Gap

The reviewed literature indicates that while CNNs achieve high performance in Pneumonia and TB classification, current state-of-the-art methods often rely on computationally expensive ensembles [10,37] or complex multi-stage segmentation pipelines [27,28]. While metaheuristics have shown promise in isolated tasks [13,19], there is a lack of comprehensive studies that: (1) address the three-class problem (Normal/Viral Pneumonia/TB) simultaneously under a unified protocol; (2) systematically compare multiple bio-inspired optimizers (WOA, GWO, CS) to tune a unified lightweight architecture; and (3) validate the results with both rigorous statistical methods (*k*-fold) and visual explainability (Grad-CAM++). This study aims to bridge this gap by proposing a metaheuristic-optimized CNN that balances high diagnostic sensitivity with computational efficiency and clinical interpretability.

## 3. Background

This section provides a comprehensive overview of the foundational technologies and concepts central to our proposed framework. We first discuss the architecture of Convolutional Neural Networks (CNNs), detailing their core components and the paradigm of Transfer Learning in the context of medical imaging. Subsequently, we introduce the principles of metaheuristic optimization, providing a theoretical justification for the selection of the specific algorithms employed in this study based on the “No Free Lunch” theorem. We also detail the validation mechanisms: Explainable AI (XAI) and statistical robustness via *k*-fold. Finally, we provide a theoretical grounding for the performance metrics selected to evaluate diagnostic reliability.

### 3.1. Convolutional Neural Networks (CNNs)

A Convolutional Neural Network (CNN) is a specialized class of deep learning models designed to automatically and adaptively learn spatial hierarchies of features from grid-like data, such as images [37]. Their architecture is inspired by the human visual cortex, making them exceptionally effective for computer vision tasks. In medical imaging, CNNs have become a cornerstone technology, enabling the development of robust systems for disease detection, classification, and segmentation [8,15]. Unlike traditional machine learning methods that rely on manual feature extraction, CNNs optimize the filters themselves during training, allowing them to capture patterns such as subtle ground-glass opacities in pneumonia that may be imperceptible to the human eye but are diagnostically relevant.

A typical CNN is composed of several key layers:

- Convolutional Layer: This is the core building block of a CNN. It performs a convolution operation on the input data by sliding a set of learnable filters (or kernels) across the image. Each filter is designed to detect a specific low-level feature, such as an edge, a color, or a texture. By stacking these layers, the network can learn to recognize more complex, high-level features (e.g., anatomical shapes or pathological patterns) in subsequent layers [8]. Mathematically, this operation generates feature maps that highlight the presence of detected features at different spatial locations, preserving the structural relationship between pixels which is vital for identifying localized abnormalities.
- Activation Function (ReLU): Following convolution, a non-linear activation function is applied. The Rectified Linear Unit (ReLU) is the standard choice, defined as f(x)=max(0,x). It introduces non-linearity to the network, allowing it to learn complex boundaries between classes and mitigating the vanishing gradient problem common in earlier sigmoid-based networks.
- Pooling Layer (Sub-sampling): The pooling layer’s function is to progressively reduce the spatial size (width and height) of the feature maps, which helps to decrease the number of parameters and computational complexity. This process, also known as downsampling, makes the feature representations more robust to small translations and distortions in the input image [8]. The most common operation, Max Pooling, retains the most prominent features while discarding irrelevant background noise.
- Fully Connected Layer: After several convolutional and pooling layers, the high-level features are flattened into a one-dimensional vector and fed into one or more fully connected layers. These layers perform the final classification task by learning non-linear combinations of the extracted features and mapping them to the output classes [38]. A softmax activation function is typically used in the final layer for multi-class classification to output a probability distribution over the classes (e.g., Normal, Viral Pneumonia, Tuberculosis).

### 3.2. Transfer Learning and Training Challenges

While powerful, the performance of a CNN is highly sensitive to its architectural and training hyperparameters. Furthermore, training deep CNNs from scratch requires massive annotated datasets, which are scarce in the medical domain. To address this, Transfer Learning has emerged as a standard strategy. This approach leverages weights from models pre-trained on large-scale datasets (e.g., ImageNet) to extract general visual features, which are then fine-tuned for the specific medical task [20,21].

However, even with Transfer Learning, the process of finding an optimal configuration specifically hyperparameters such as learning rate, batch size, dropout rate, and momentum remains a complex optimization problem. Improper tuning can lead to overfitting where the model memorizes the training data but fails to generalize to unseen X-rays, or vanishing gradient issues that stall learning [30]. These challenges motivate the use of advanced, automated search strategies beyond simple trial-and-error.

### 3.3. Metaheuristic Optimization

Metaheuristic algorithms are high-level, stochastic optimization techniques inspired by natural phenomena. They are particularly effective for solving complex, non-differentiable, and high-dimensional optimization problems where traditional methods often fail.

Because the search space for Deep Learning HPO is complex and non differentiable, traditional gradient based and exhaustive search methods often fail [31]. Consequently, there is a growing shift towards swarm intelligence in medical CNN optimization, driven by its superior capability to navigate high dimensional landscapes efficiently [23]. Recent literature highlights that hybrid metaheuristic approaches effectively tune critical hyperparameters, such as learning rates and convolution kernels, improving both training efficiency and diagnostic accuracy in complex clinical scenarios [39].

For this study, the selection of Cuckoo Search (CS), Grey Wolf Optimizer (GWO) and Whale Optimization Algorithm (WOA) is based on the results of a preliminary comparative study conducted by our team. In that analysis, CS, GWO, and FA consistently demonstrated the most effective performance. Furthermore, this selection is theoretically grounded in the “No Free Lunch” (NFL) theorem [40], which states that no single optimization algorithm performs best for all classes of problems. Therefore, it is scientifically necessary to evaluate algorithms with distinct search logic: the heavy-tailed exploration of CS, the spiral exploitation of WOA, and the hierarchical leadership of GWO.

#### 3.3.1. Cuckoo Search (CS)

Inspired by cuckoos laying eggs in other birds’ nests, CS explores the solution space using a mix of local and global search strategies. The global search uses Lévy flights, which involve random jumps with heavy-tailed probability distributions. This allows the algorithm to cover the search space more efficiently than standard Gaussian walks, making it exceptionally robust at escaping local minima during the hyperparameter tuning process [40,41].

#### 3.3.2. Whale Optimization Algorithm (WOA)

WOA is inspired by the bubble-net hunting strategy of humpback whales. While algorithms like CS utilize Lévy flights for global exploration, WOA’s strength lies in its unique spiral movement, which provides exceptional precision in refining sensitive parameters like the learning rate [42,43]. To address the complex error landscapes inherent in Tuberculosis detection, characterized by shallow local minima and overlapping pathological patterns, our framework leverages the exploration-exploitation balance of WOA, which has recently outperformed Genetic Algorithms and PSO in medical diagnostic robustness [44] Furthermore, the efficiency of WOA in lung disease contexts is supported by its successful application in tuberculosis-specific sensor and feature optimization, achieving sensitivities exceeding 98% [45]. To mitigate premature convergence, we consider advanced mechanisms such as altruistic search behaviors, ensuring that candidate solutions with global potential survive across iterations even if they exhibit temporary sub-optimal fitness [46].

#### 3.3.3. Grey Wolf Optimizer (GWO)

GWO emulates the social hierarchy of a wolf pack. The three best solutions (alpha, beta, delta) guide the search, while others (omega) follow. This hierarchy naturally favors strong exploitation, ensuring fast convergence towards the global optimum once the search has narrowed down to a promising region [47].

### 3.4. Explainable AI (XAI) and Grad-CAM++

Despite the high accuracy of CNNs, their “black-box” nature poses a significant barrier to clinical adoption. In medical diagnostics, it is insufficient for a model to merely output a prediction; it must also provide visual evidence to verify that it is focusing on relevant pathological markers rather than confounding artifacts [12].

To address this, we employ Gradient-weighted Class Activation Mapping (Grad-CAM) and its enhanced version, Grad-CAM++. This technique utilizes the gradients of the target concept (e.g., ”Viral Pneumonia”) flowing into the final convolutional layer to produce a coarse localization map highlighting the important regions in the image [36]. Grad-CAM++ improves upon the original by providing better localization of objects and capturing multiple occurrences of the same class [15]. In our framework, this serves as a qualitative validation step: a classification is only considered reliable if the heatmap overlaps with radiological findings.

### 3.5. Statistical Validation: k-Fold Cross-Validation

Medical imaging datasets often suffer from limited size and class imbalance. To ensure statistical robustness and mitigate overfitting, we implement *k*-Fold Cross-Validation. In this protocol, the dataset is randomly partitioned into *k* equal subsamples. The model is trained *k* times, using a different fold as the validation set each time. The final performance metric is the average of the results [1]. This method ensures that every image is used for both training and validation, providing an unbiased estimate of the model’s generalization ability [17].

### 3.6. Performance Evaluation Metrics

In medical imaging classification, while Accuracy provides a general indicator of performance, it is necessary to utilize a multi-metric approach to ensure a comprehensive evaluation of the diagnostic model. The following metrics, derived from the Confusion Matrix, are employed to assess the proposed hybrid framework:

- Accuracy: The ratio of correctly predicted observations to the total observations. Since the experimental dataset is perfectly balanced across all classes, Accuracy is utilized as the primary objective function to guide the metaheuristic search process toward global convergence.
- Recall (Sensitivity): The ratio of correctly predicted positive observations to all observations in the actual class. This metric quantifies the model’s ability to identify positive cases of Tuberculosis and Viral Pneumonia, which is essential for minimizing the occurrence of False Negatives during the diagnostic process.
- F1-Score: The harmonic mean of Precision and Recall. This metric provides a balanced measure of the model’s diagnostic robustness, representing the trade-off between sensitivity and precision across the different pathological classes evaluated in this study.

### 3.7. Methodological Robustness and Sensitivity Analysis

To ensure that the model’s performance is independent of specific parameter initializations, a comprehensive sensitivity analysis is integrated into the experimental protocol [48]. This evaluation systematically examines the influence of population size and metaheuristic control parameters on the final F1-score, verifying the stability of the optimized configurations across diverse search conditions. Statistical rigor is maintained through the application of non-parametric assessments, specifically the Friedman and Wilcoxon signed-rank tests, to validate the consistency of the findings. Furthermore, the inclusion of ablation studies allows for the isolation of individual architectural components’ contributions, thereby establishing the clinical reliability and diagnostic robustness required for effective respiratory disease screening [49,50].

This multi-layered validation strategy ensures that the proposed hybrid model achieves a balance between high diagnostic performance and computational consistency.

## 4. Methodology

This section details the structured framework designed to develop and evaluate a metaheuristic-optimized Convolutional Neural Network (CNN) for the automated differential diagnosis of viral pneumonia and tuberculosis from chest X-ray (CXR) images. The methodology encompasses data acquisition and preprocessing, the hybrid optimization framework, and the experimental setup for robust model evaluation.

Artificial Intelligence Disclosure: The Gemini 3 Flash large language model (Google DeepMind, Mountain View, CA, USA) was employed as a support tool for synthesizing complex concepts and organizing the manuscript’s structure. This tool was used to enhance technical clarity and ensure narrative consistency. All outputs were strictly reviewed, verified, and edited by the authors to maintain academic rigor and accuracy.

### 4.1. Proposed Hybrid Approach

**Figure 1:**
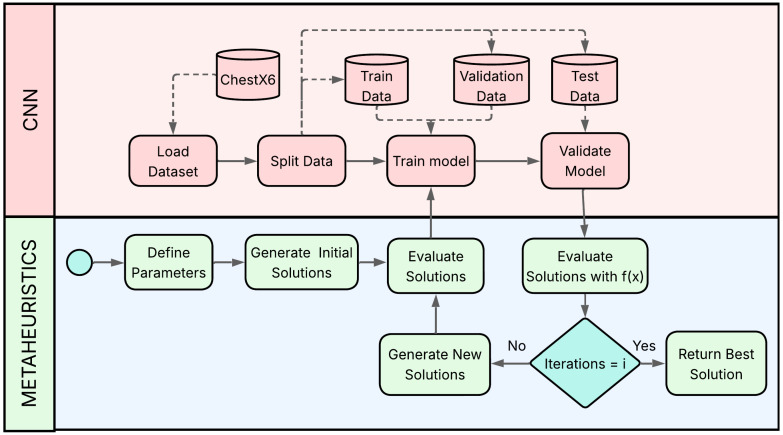
Hybrid proposal CNN-MHs.

The hybrid optimization strategy proposed in this study aims to enhance the classification performance of CNNs by incorporating metaheuristic algorithms into the hyperparameter tuning process. This approach seeks to address the shortcomings of conventional optimization techniques when dealing with the high-dimensional, nonlinear, and multimodal search spaces typical of deep learning architectures. CNNs are highly sensitive to design parameters such as the number of convolutional layers, kernel sizes, learning rate, and batch size [30,31]. Manual tuning and grid search methods are often inefficient and computationally demanding [30]. In contrast, metaheuristics bio-inspired algorithms modeled after natural processes such as swarm intelligence, foraging behavior, or evolutionary mechanisms offer a promising alternative for exploring complex solution spaces with fewer assumptions and stronger global search capabilities [31,51]. The conceptual framework is illustrated in Figure 1, which highlights the collaborative interaction between the CNN model and the metaheuristic engine. The process begins with loading and partitioning the dataset for model training and evaluation. Simultaneously, the metaheuristic algorithm generates an initial population of candidate hyperparameter configurations. These candidates are iteratively assessed using a predefined objective function (e.g., classification accuracy), and improved solutions are derived through guided search strategies. The CNN model is retrained and validated continuously within this evaluation loop, allowing its configuration to adapt dynamically.

We propose a hybrid framework that synergizes the feature extraction power of CNNs with the advanced search capabilities of metaheuristic algorithms. The core objective is to automate the discovery of an optimal set of CNN hyperparameters, a task that is often intractable with manual tuning or grid search methods. The optimization process follows an iterative loop:

1. A metaheuristic algorithm (CS, GWO, or WOA) initializes a population of candidate solutions, where each solution represents a complete set of CNN hyperparameters.
2. For each candidate solution, a CNN model is dynamically constructed and compiled with the specified hyperparameters.
3. The constructed CNN is trained and validated on the dataset.
4. The performance of the trained CNN is evaluated using a predefined objective function. This performance value serves as the “fitness” of the candidate solution.
5. The fitness value is fed back to the metaheuristic algorithm, which then uses its specific update rules (e.g., Lévy flights for CS, wolf pack hunting for GWO) to generate a new population of improved candidate solutions for the next iteration.
6. This loop continues until a termination criterion is met (e.g., a maximum number of iterations or convergence of the fitness score), yielding a final, optimized set of hyperparameters.

The overall optimization workflow of the proposed hybrid framework is summarized in Algorithm 1. The algorithm describes the iterative interaction between the CNN model and the metaheuristic optimization process, including population initialization, dynamic CNN construction, fitness evaluation, and hyperparameter updating until convergence is achieved.


**Algorithm 1** Hybrid Framework for CNN Optimization via Metaheuristics1: **Input:** ChestX6 Dataset (Train/Val splits), Search Space (*D*),
   Maximum Iterations (*T*), Population Size (*N*). 2: **Output:** Optimal hyperparameter set ($X_{best}$) and trained CNN model. 3: **Initialization:** Generate a population of *N* agents $X_{i}$ within the bounds of *D*. Each agent represents the vector: [num_layers,num_filters,batch_norm,epochs,batch_size,lr]. 4: **for** each agent $X_{i}$ in the initial population **do** 5:     Dynamically construct $CNN_{i}$ architecture according to the parameters in $X_{i}$. 6:     Train $CNN_{i}$ using the training set. 7:     Evaluate $Fitness(X_{i})=\mathrm{Accuracy}$ using the validation set (Equation (1)). 8: **end for** 9: Set $X_{best}=\mathrm{Agent}\;\mathrm{with}\;\mathrm{the}\;\mathrm{highest}\;\mathrm{Fitness}$. 10: **while** 
t≤T 
**do** 11:   **for** each agent $X_{i}$ **do** 12:      Update the position of $X_{i}$ following the specific metaheuristic logic: 13:         **WOA:** Spiral movement or bubble-net encircling. 14:         **GWO:** Social hierarchy (Alpha, Beta, Delta wolves). 15:         **CS:** Lévy flights and nest replacement. 16:      Apply boundary conditions to maintain $X_{i}$ within the search space *D*. 17:   **end for** 18:   **for** each updated agent $X_{i}$ **do** 19:      Re-construct and re-train the $CNN_{i}$ architecture with the new parameters. 20:      Calculate the new $Fitness(X_{i})$. 21:      **if** $Fitness\left(X_{i}\right)>Fitness\left(X_{best}\right)$ **then** 22:         Update the global best: $X_{best}=X_{i}$. 23:      **end if** 24:   **end for** 25:   t=t+1 26: **end while** 27: **return** $X_{best}$ and the optimal CNN configuration.


### 4.2. CNN Architecture

Unlike approaches that rely on heavy, pre-trained Transfer Learning models (such as ResNet or DenseNet), this study deliberately utilizes a custom, lightweight CNN built from scratch. This design choice is fundamental to the proposed methodology: utilizing pre-trained models with rigid topologies would restrict the metaheuristic search space merely to final-stage hyperparameters. Conversely, a custom CNN provides the structural malleability required for the WOA, GWO, and CS algorithms to dynamically alter deep architectural traits (e.g., convolutional depth and filter counts). Furthermore, embedding massive pre-trained networks inside a population-based iterative optimization loop is computationally prohibitive. Thus, our goal is to prove that a dynamically optimized simple architecture can match or exceed the diagnostic efficacy of heavy models while retaining the low computational footprint (FLOPs) required for real-world clinical deployment.

**Figure 2:**
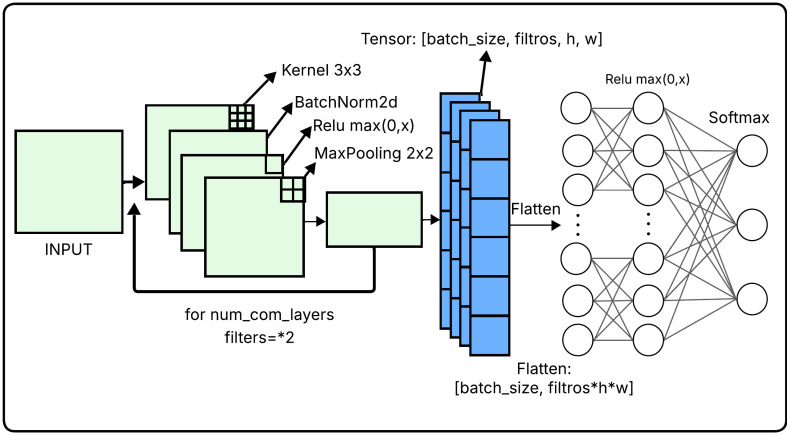
Architecture of CNN.

The baseline CNN architecture employed in this study Figure 2 follows a modular and adaptable design to accommodate varying depths and filter configurations during optimization. Table 1 provides a summary of the core architectural components, detailing their role and implementation within the model pipeline.

### 4.3. Objective Function and Evaluation Metrics

The objective function used to guide the metaheuristic optimization process is classification Accuracy:

(1) $$max\;f\left(X\right)=Accuracy=\frac{TP+TN}{TP+TN+FP+FN}$$

where TP, FP, TN, and FN represent true positives, false positives, true negatives, and false negatives, respectively. In this study, the choice of Accuracy as the primary objective (function fitness function) is technically justified by the creation of a perfectly balanced dataset. This pre-balancing step ensures that the optimization engine can achieve mathematical convergence and general model stability during the training phase without being biased by class distribution.

However, while Accuracy effectively guides the metaheuristic search, it is insufficient for clinical validation. Following established standards in high-impact medical AI research [49], Recall (Sensitivity) and Specificity are established as the imperative metrics for clinical reporting. In the context of infectious diseases like Tuberculosis and Viral Pneumonia, maximizing Recall ensures clinical safety by strictly minimizing False Negatives (missed critical cases). Thus, the framework utilizes Accuracy to guarantee robust algorithmic convergence, while relying on Recall and Specificity to validate the model’s diagnostic reliability. Additionally, F1-Score and AUC are reported to provide a robust summary of the classifier’s discrimination capacity.

To obtain a comprehensive performance assessment, additional metrics are reported: Recall, Specificity, F1-Score, and AUC, as detailed in Table 2.

This experimental design enables a robust and systematic evaluation of the hybrid CNN-MH framework across varying optimization schemes and parameter configurations. By combining clinical relevance with statistical rigor, the assessment integrates key metrics such as Recall, Specificity, F1-Score, and AUC that capture different dimensions of diagnostic model performance.

As outlined in Table 2, Recall quantifies the model’s sensitivity to positive viral pneumonia and tuberculosis cases, critical for reducing false negatives. Specificity evaluates the ability to avoid false positives. F1-Score offers a balanced measure under class imbalance by combining precision and recall, while AUC summarizes model discrimination across decision thresholds [52]. This metric set has proven effective for evaluating classifiers in medical AI contexts [53,54], ensuring a reliable basis for comparing metaheuristic driven configurations.

### 4.4. Dataset

The dataset used in this work is a repository of chest X-ray images, organized for multi-class classification of pulmonary diseases. It is structured into three main folders /train, /val, and /test, corresponding to model training, validation, and final test evaluation, respectively. Each of these folders contains six subdirectories, one per disease class.

In total, the collection includes 18,036 grayscale images, all resized to 224×224 pixels. The images originate from a combination and reorganization of multiple publicly available chest X-ray repositories, with the goal of building a balanced and diverse dataset for multi-class classification of respiratory conditions.

Table 3 reports the number of images in the training, validation, and test splits for each class.

This dataset was created by combining and reorganizing publicly available chest X-ray data from multiple repositories, including images of viral pneumonia, COVID-19, and tuberculosis, as well as the COVID, Bacterial, Viral, Normal, Emphysema Dataset. By integrating these sources, we constructed a diverse and balanced collection tailored for multi-class classification of respiratory conditions.

Although the original dataset comprises six classes, this study focuses on only three of them: Normal, Tuberculosis and Pneumonia-Viral. These classes were selected to center the experiments on distinguishing healthy cases, chronic bacterial infections (tuberculosis), and viral pneumonia. This three-class subset was selected to ensure a balanced and controlled experimental setting focused on a clinically relevant differential diagnosis scenario. However, additional respiratory conditions available in ChestX6, such as COVID-19 and bacterial pneumonia, were not included in the present experimental scope. This subset forms the basis for training and evaluating the proposed models.

### 4.5. Computational Complexity Analysis

The computational cost of this hybrid approach is overwhelmingly dominated by the CNN training, not the metaheuristic’s internal operations. The theoretical complexity for each iteration can be described as $O(N\times D+N\times C_{CNN})$, where *N* is the population size, *D* is the number of hyperparameters to optimize, and $C_{CNN}$ is the cost of training a single CNN model. Since the cost of training the network ($C_{CNN}$) is significantly greater than the algorithm’s own operations, the overall complexity for *T* iterations simplifies to $O(T\times N\times C_{CNN})$. As all six algorithms in this study share this fundamental structure, their theoretical complexities are comparable. The practical differences in observed runtimes stem from how efficiently each search strategy converges, which can influence the final training parameters found (such as epochs) and thus the actual cost of $C_{CNN}$.

### 4.6. Model Interpretability with Grad-CAM++

To enhance model transparency and support human +++AI decision making, we adopt Grad-CAM++ as a visual explanation tool. Grad-CAM++ extends the original gradient-weighted class activation mapping approach and can produce class-discriminative saliency maps from any convolutional layer of a CNN [55,56].

The method computes a weighted combination of the feature maps in a chosen convolutional layer, where the weights are obtained from the gradients of the target class score with respect to each activation. Compared to Grad-CAM, the “++” variant incorporates higher-order gradient information, yielding sharper and more localized heatmaps useful when multiple findings may overlap or visual differences are subtle.

Formally, for class *c*, the saliency map $L_{\mathrm{Grad}\text{-}\mathrm{CAM}++}^{c}$ is given by

$$L_{\mathrm{Grad}\text{-}\mathrm{CAM}++}^{c}=\mathrm{ReLU}\;\left(\sum_{k}\alpha_{k}^{c}\;A^{k}\right),$$

where $A^{k}$ denotes the *k*-th activation map of the selected layer and $\alpha_{k}^{c}$ are weights derived from first- and higher-order derivatives of the class score $y^{c}$ with respect to the activations $A_{ij}^{k}$ (e.g., terms such as $\frac{\partial^{2}y^{c}}{\partial {\left(A_{ij}^{k}\right)}^{2}}$).

By overlaying the resulting heatmaps on the original chest X-ray images, Grad-CAM++ highlights the regions that most influenced the model’s prediction for pulmonary disease classes (Normal, Viral Pneumonia, and Tuberculosis). This improves interpretability and provides clinically meaningful cues for expert verification, which is crucial in medical imaging applications where accountability and user trust are essential.

### 4.7. Statistical Validation Protocol

To assess whether the observed performance differences between the optimized classifiers were statistically meaningful, pairwise comparisons were conducted using the Wilcoxon signed-rank test [57] over the key diagnostic metric: Accuracy. Each comparison involved a one-sided test, evaluating whether each metaheuristic-optimized convolutional architecture (CNN-WOA, CNN-GWO, or CNN-CS) significantly outperformed the baseline CNN model across the independent cross-validation folds (5 and 10 folds). To ensure the robustness and reliability of our findings, a rigorous validation strategy will be employed. We will use *k*-fold cross-validation (with *k* = 5 and 10) during the evaluation phase. This technique helps to ensure that the performance results are not dependent on a single, arbitrary train–test split and provides a better estimate of the model’s generalization ability on unseen data [15]. Furthermore, to compare the performance distributions of the final models optimized by CS, GWO, and WOA against the baseline, a non-parametric statistical test will be used to determine if the differences in their performance are statistically significant. We will use the Wilcoxon signed-rank test. This test is specifically chosen for two reasons: (1) It is non-parametric, making it suitable for small sample sizes like k=5 or k=10 folds where a normal distribution cannot be assumed. (2) It is designed for paired samples, which directly matches our experimental design. The performance of each model on a given test fold (e.g., Base on Fold 1) is paired against the performance of another model on the exact same test fold (e.g., CS on Fold 1), creating a paired comparison.

Furthermore, to ensure statistical robustness, 95% Confidence Intervals (CIs) were computed for all performance metrics using the Student’s t-distribution with N−1 degrees of freedom. The upper bounds of the intervals were strictly capped at 1.0 to preserve the mathematical coherence of the probability-based metrics.

The dual approach of evaluating the models under both 5-fold and 10-fold cross-validation was purposefully designed. The 5-fold protocol establishes a standard performance baseline. Conversely, the 10-fold protocol serves two critical functions: first, it acts as a rigorous stress test to evaluate the architectural stability under higher data fragmentation; second, it is mathematically necessary to provide an adequate sample size (N=10) for valid non-parametric hypothesis testing, as the two-sided Wilcoxon signed-rank test is mathematically constrained to a minimum *p*-value of 0.0625 when using only 5 folds, precluding the confirmation of statistical significance at the α=0.05 level.

### 4.8. Hypothesis Test

To determine if the observed differences are statistically significant, a formal hypothesis test is established.

The Null Hypothesis ($H_{0}$) postulates that there is no statistically significant difference in the performance metric between the CNN-Base model and an optimized model (CNN + MH):

$$H_{0}:\mu_{\mathrm{Base}}=\mu_{\mathrm{MH}}$$

The Alternative Hypothesis $H_{1}$ postulates that there is a statistically significant difference between the performance of the CNN-Base model and the optimized model:

$$H_{1}:\mu_{\mathrm{Base}}\neq \mu_{\mathrm{MH}}$$

The Wilcoxon signed-rank test is used (as previously mentioned), and a significance level (α) of 0.05 is set. If the resulting *p*-value is less than α, ($H_{0}$) will be rejected in favor of ($H_{1}$).

## 5. Experiments

### 5.1. Environment

The experiments were carried out in a Python version 3.11.15 (Python Software Foundation, Wilmington, DE, USA) based computational environment, using the capabilities of two clusters of the Pontificia Universidad Católica de Valparaíso (PUCV), one belonging to the School of Computer Engineering equipped with the Omoikane machines, equipped with a NVIDIA RTX 4090 GPU (24 GB) and the Grace machine, equipped with a NVIDIA RTX 4070 Ti SUPER GPU (16 GB). The Convolutional Neural Network (CNN) models were implemented and trained using the PyTorch framework (Meta AI, Menlo Park, CA, USA), specifically version 2.5.1, along with the Torchvision library version 0.20.1 for handling image datasets and transformations.

### 5.2. Experimental Dataset

Table 4 presents the distribution of images used in the experiments for the three selected classes.

**Figure 3:**
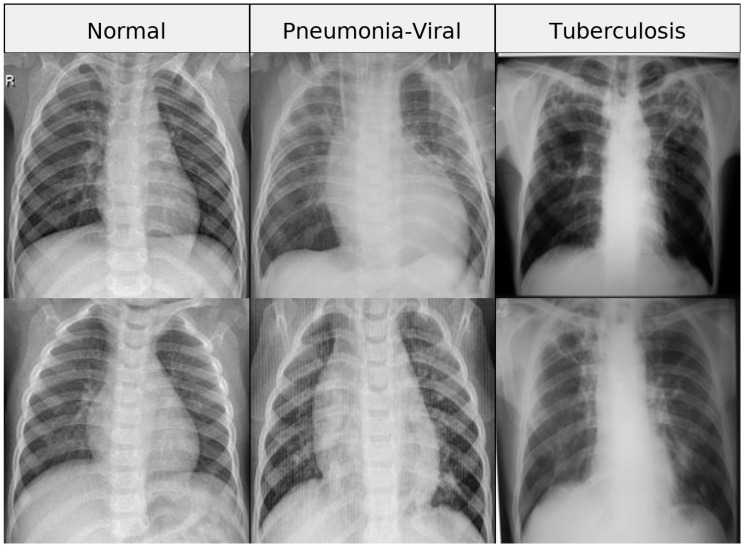
Examples of chest X-rays by class: Normal (**left column**), Viral pneumonia (**center column**), and Tuberculosis (**right column**).

To complement the dataset distribution presented in Table 4, Figure 3 provides a compact visual overview of representative chest X-ray samples from each class (Normal, Viral Pneumonia, and Tuberculosis). This figure offers an intuitive understanding of the visual characteristics and variability within the dataset, as well as the inherent complexity of the classification task.

In addition to providing representative samples, Figure 3 enables a qualitative visual analysis of the dataset. As observed, normal cases exhibit clear lung fields with well-defined anatomical structures, while viral pneumonia cases show diffuse opacities and interstitial patterns. In contrast, tuberculosis images present more localized abnormalities, including consolidations and structural distortions. These visual differences highlight the intrinsic complexity of the classification task, particularly due to overlapping patterns between viral pneumonia and tuberculosis, thereby justifying the need for robust and discriminative models.

### 5.3. Hyperparameter Tuning

The base CNN architecture is designed to be flexible, allowing its structure to be modified by the metaheuristic optimizers. It consists of a variable number of convolutional blocks followed by a classification head. Each block contains a convolutional layer, an optional batch normalization layer, and a max-pooling layer. The set of hyperparameters that define the search space for the metaheuristics is shown in Table 5:

### 5.4. Metaheuristic Parameters and Schemes

The experiments were conducted using a single optimization scheme for all three metaheuristics (GWO, CS, and WOA), defined by a specific population size (Pop. size) and a corresponding number of iterations (iter), which served as the termination criterion. These parameters govern the algorithm’s search capacity and convergence behavior across the solution space.

The population was fixed at 10 agents and the maximum iterations were set to 10. This compact configuration aligns with the rapid convergence behavior observed in recent successful medical applications of swarm intelligence [23], demonstrating that algorithms like WOA can effectively navigate complex pathological feature spaces without requiring massive iterations to escape local optima. This efficient parametric approach is further supported by foundational settings established in related computer vision optimizations [58,59]. This single scheme was used for all experimental runs, applied to both the 5-fold and 10-fold validation protocols, as summarized in Table 6.

To establish a rigorous performance benchmark and quantify the specific contribution of the metaheuristic optimization, a control model termed CNN-Base was implemented [30]. This model utilizes the same architectural framework described in Section 3.3 but employs a fixed set of hyperparameters selected manually based on standard deep learning practices and empirical experience [60]. By comparing the optimized variants (CNN-WOA [42], CNN-GWO [47], CNN-CS [22]) against this manual baseline, the study aims to isolate and validate the improvements in sensitivity and accuracy solely attributable to the automated search process [16].

### 5.5. Baseline Configuration

To evaluate the effectiveness of the metaheuristic optimization, the CNN-Base model was trained using a fixed, manually selected configuration derived from standard practices in medical image classification. This baseline architecture consists of a lightweight structure of 4 convolutional layers with 128 filters per layer, aligning with findings that simple and lightweight CNN structures can yield more satisfactory results and avoid overfitting when dealing with limited medical datasets. Furthermore, the model utilizes Batch Normalization to improve training convergence and ensure stability. The training process was conducted using a learning rate of 0.001, a batch size of 128, and a total of 20 epochs. These parameters serve as the control variable to quantify the performance and efficiency gains achieved by the proposed hybrid optimization schemes, departing from conventional methods that rely strictly on trial and error for predefined structures [61].

## 6. Results and Discussion

This section presents the experimental results obtained from the 5-fold cross-validation. The analysis compares the performance of the hybrid CNN models optimized via metaheuristics (WOA, GWO, and CS) against a baseline (CNN-Base) with manually configured hyperparameters. The objective is to evaluate whether metaheuristic optimization offers a statistically significant and robust improvement in diagnostic metrics, with a special emphasis on clinical sensitivity.

### 6.1. Quantitative Performance Metrics

Table 7 summarizes the average model performance from the 5-fold cross-validation. The mean and standard deviation (AVG ± STD) are reported for each model across the key evaluation metrics.

The quantitative analysis reveals a decisive advantage for the hybrid optimization approach. Regardless of the validation protocol employed, whether 5-fold or 10-fold, the models optimized via metaheuristics consistently surpassed the baseline model across all diagnostic dimensions. This suggests that the hyperparameter configuration found manually, despite following standard practices, resided in a suboptimal region of the search space, leading to lower generalization capability.

In the 5-fold cross-validation scenario, the CNN-WOA model demonstrated superior overall efficacy, particularly in correctly identifying negative cases, as evidenced by a specificity of 99.02%. This high specificity is crucial in clinical screening to reduce false positives and avoid unnecessary anxiety or follow-up procedures for healthy patients. While the CNN-GWO model achieved a marginally higher recall (98.34%) compared to CNN-WOA (98.08%), the difference is minimal. However, contrasting these with the baseline is significant; the manual model only achieved a recall of 94.45%. In a clinical context involving infectious diseases like tuberculosis and viral pneumonia, a sensitivity gap of nearly 4% represents a substantial risk of missed diagnoses, reinforcing the necessity of the optimization process.

When subjecting the models to the more rigorous 10-fold validation, which increases data fragmentation and tests stability more intensively, the robustness of CNN-WOA became even more pronounced. Unlike the 5-fold scenario where results were mixed, in the 10-fold tests, CNN-WOA achieved the highest scores across all metrics, including Recall (98.18%) and F1-Score (98.19%). This indicates that the Whale Optimization Algorithm is particularly effective at finding architectural configurations that generalize well even when training data is partitioned into smaller subsets.

Table 8 presents the comprehensive performance evaluation of the models across 5-fold and 10-fold cross-validation scenarios, detailing the mean, standard deviation, and 95% Confidence Intervals (CI). Analyzing the confidence intervals provides crucial insights into the true generalization capabilities of the architectures. Across both validation protocols, the metaheuristic-optimized models (WOA, GWO, and CS) consistently demonstrate not only higher mean metrics but also significantly narrower CIs compared to the baseline (CNN-Base). For instance, in the 10-fold scenario, the CNN-WOA model achieves an Accuracy CI of [0.985, 0.991], indicating highly stable performance regardless of the specific data partition. In contrast, the CNN-Base model exhibits a wider CI [0.967, 0.977] and a higher standard deviation, reflecting a concerning sensitivity to data fragmentation.

This stability is particularly vital regarding Recall, a critical metric for patient safety in medical diagnostic models where minimizing false negatives is paramount. The CNN-WOA model exhibits an exceptionally tight Recall CI of [0.981, 0.983] under 10-fold cross-validation. This narrow margin guarantees a consistently high true positive rate even in the worst-case statistical scenario, vastly outperforming the CNN-Base, whose lower bound drops dangerously to 0.918. Ultimately, this tight variance confirms that the structural optimization provided by the metaheuristic algorithms successfully mitigates the risk of overfitting and ensures robust, reliable diagnostic capabilities suitable for clinical environments.

### 6.2. Sensitivity and Robustness Analysis

To evaluate the impact of the stochastic optimization process and the model’s robustness, we analyze the performance variability across the cross-validation folds. The low standard deviation (STD) reported in Table 7 for the CNN-WOA model (±0.30 and ±0.41 in accuracy for 5 and 10 folds, respectively) indicates a low sensitivity to the initial data distribution. To further analyze the contribution of individual design components, an ablation-style analysis is presented in Table 9, summarizing the impact of metaheuristic optimization on model performance.

As shown in Table 9, the ablation-style analysis indicates that metaheuristic optimization consistently improves all evaluation metrics compared to the baseline CNN, highlighting its central role in performance enhancement. All optimized variants outperform the manually configured model, demonstrating that automated hyperparameter search enables a more effective exploration of the solution space. Among the evaluated strategies, CNN-WOA achieves the best overall results, suggesting a more effective balance between exploration and exploitation.

Furthermore, the robustness of the framework is validated by the convergence of the three distinct metaheuristics (WOA, GWO, and CS) toward similar architectural regions universally selecting Batch Normalization and learning rates in the order of $10^{-4}$. This cross-algorithm consistency demonstrates that the framework effectively identifies stable, high-performing regions of the search space, ensuring that the results for CNN-WOA are a product of a robust optimization process rather than a fortunate initialization.

**Figure 4:**
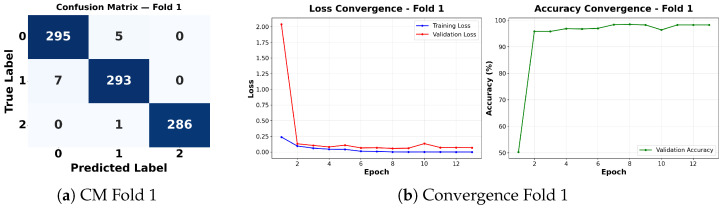
Confusion matrix and convergence curve for Fold 1 (CNN-WOA).

**Figure 5:**
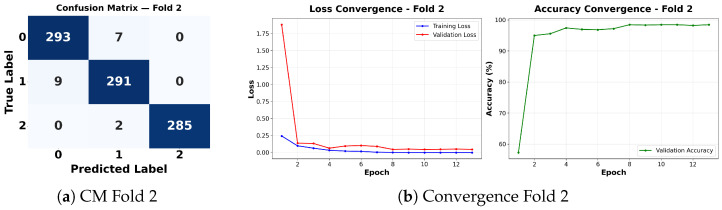
Confusion matrix and convergence curve for Fold 2 (CNN-WOA).

**Figure 6:**
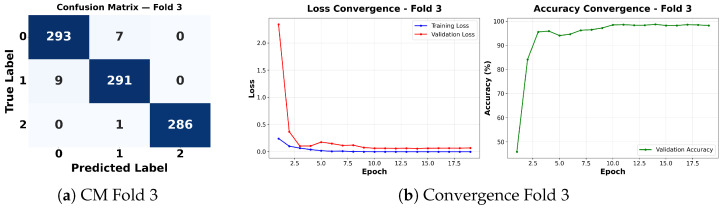
Confusion matrix and convergence curve for Fold 3 (CNN-WOA).

**Figure 7:**
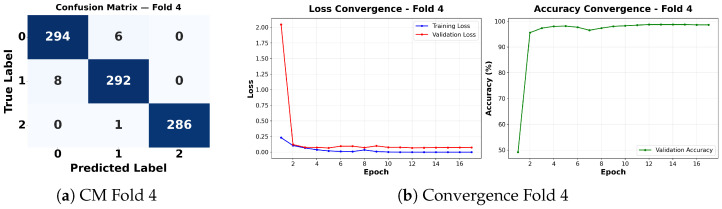
Confusion matrix and convergence curve for Fold 4 (CNN-WOA).

**Figure 8:**
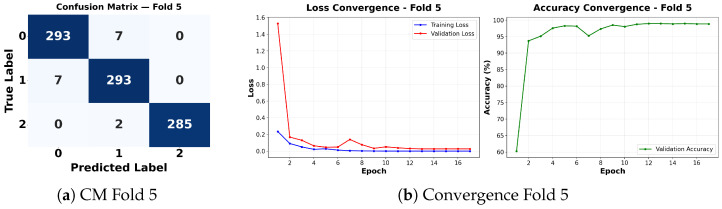
Confusion matrix and convergence curve for Fold 5 (CNN-WOA).

**Figure 9:**
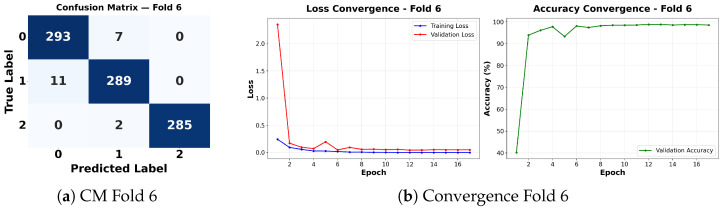
Confusion matrix and convergence curve for Fold 6 (CNN-WOA).

**Figure 10:**
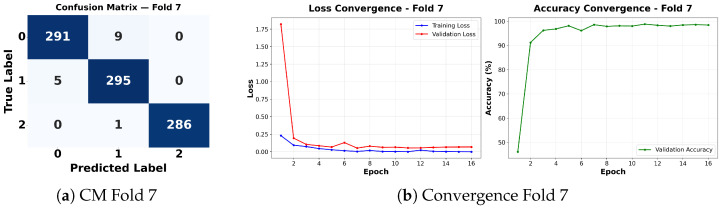
Confusion matrix and convergence curve for Fold 7 (CNN-WOA).

**Figure 11:**
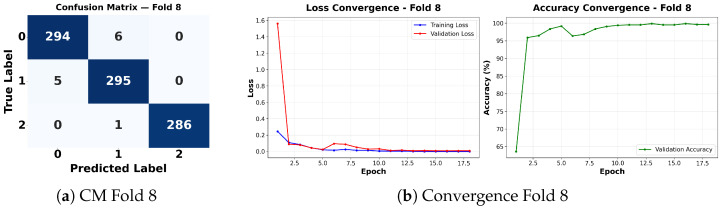
Confusion matrix and convergence curve for Fold 8 (CNN-WOA).

**Figure 12:**
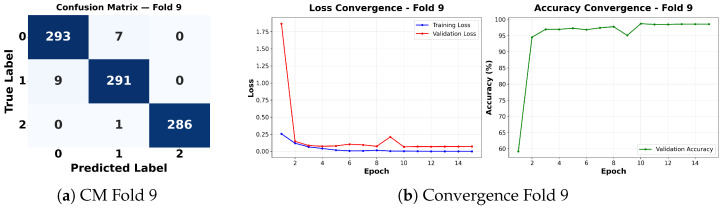
Confusion matrix and convergence curve for Fold 9 (CNN-WOA).

**Figure 13:**
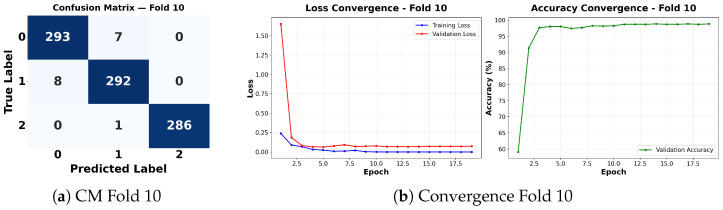
Confusion matrix and convergence curve for Fold 10 (CNN-WOA).

**Figure 14:**
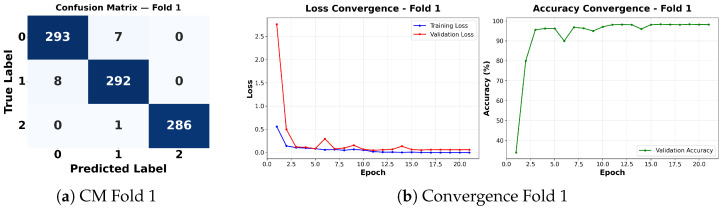
Confusion matrix and convergence curve for Fold 1 (CNN-GWO).

**Figure 15:**
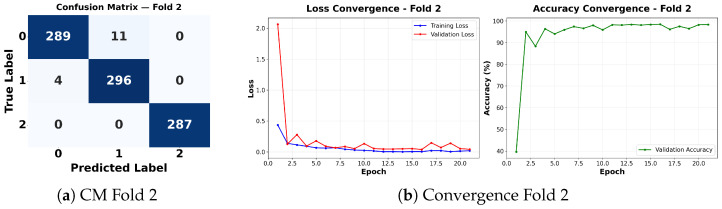
Confusion matrix and convergence curve for Fold 2 (CNN-GWO).

**Figure 16:**
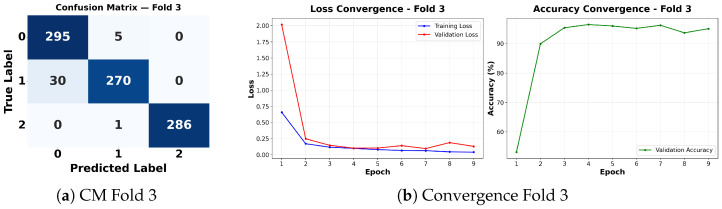
Confusion matrix and convergence curve for Fold 3 (CNN-GWO).

**Figure 17:**
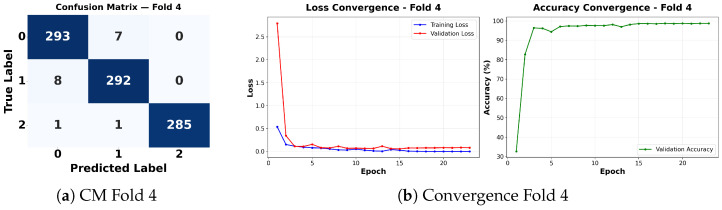
Confusion matrix and convergence curve for Fold 4 (CNN-GWO).

**Figure 18:**
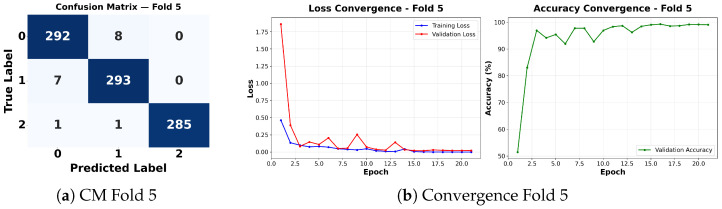
Confusion matrix and convergence curve for Fold 5 (CNN-GWO).

**Figure 19:**
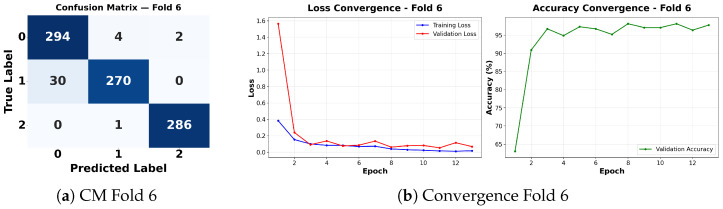
Confusion matrix and convergence curve for Fold 6 (CNN-GWO).

**Figure 20:**
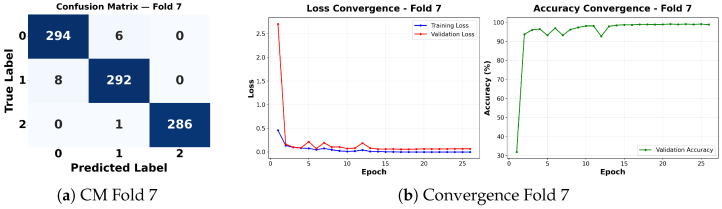
Confusion matrix and convergence curve for Fold 7 (CNN-GWO).

**Figure 21:**
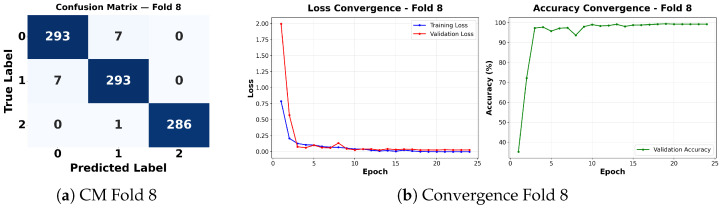
Confusion matrix and convergence curve for Fold 8 (CNN-GWO).

**Figure 22:**
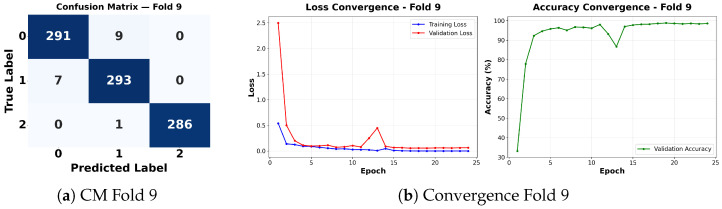
Confusion matrix and convergence curve for Fold 9 (CNN-GWO).

**Figure 23:**
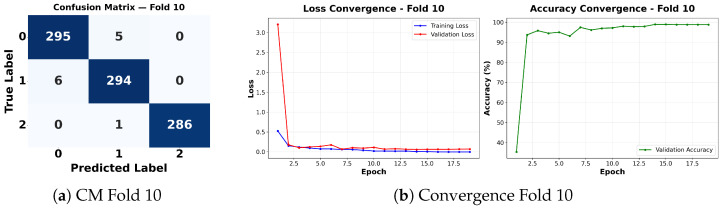
Confusion matrix and convergence curve for Fold 10 (CNN-GWO).

Complementing the aggregate metrics, the detailed fold-by-fold results for the metaheuristic models are presented through confusion matrices and convergence curves to ensure complete transparency. The confusion matrices shown in Figure 4a, Figure 5a, Figure 6a, Figure 7a, Figure 8a, Figure 9a, Figure 10a, Figure 11a, Figure 12a and Figure 13a for CNN-WOA and Figure 14a, Figure 15a, Figure 16a, Figure 17a, Figure 18a, Figure 19a, Figure 20a, Figure 21a, Figure 22a and Figure 23a for CNN-GWO confirm the strong classification capability across the three classes, while the convergence curves presented in Figure 4b, Figure 5b, Figure 6b, Figure 7b, Figure 8b, Figure 9b, Figure 10b, Figure 11b, Figure 12b and Figure 13b for CNN-WOA and Figure 14b, Figure 15b, Figure 16b, Figure 17b, Figure 18b, Figure 19b, Figure 20b, Figure 21b, Figure 22b and Figure 23b for CNN-GWO demonstrate that the optimization process efficiently guides the network toward optimal hyperparameter configurations, successfully avoiding local minima and achieving rapid stabilization. Crucially, across all folds, the validation accuracy and loss curves closely track the training metrics without diverging. This behavior provides explicit evidence that the proposed dynamically optimized architectures do not suffer from overfitting, maintaining robust generalization capability.

Furthermore, analyzing the specific fold distributions provides deeper clinical insights into the model’s reliability. Notably, the fourth fold of the CNN-WOA model, Figure 17a achieved the highest performance across all experimental runs, representing the peak diagnostic capability of the proposed system. In this best-performing scenario, the architecture demonstrates a remarkable diagnostic balance with minimal class confusion, correctly identifying 294 out of 300 Normal cases (Class 0) and 292 out of 300 Viral Pneumonia cases (Class 1). Most notably, the model exhibited exceptional sensitivity in detecting Tuberculosis (Class 2), successfully classifying 286 out of 287 cases. This near-perfect Recall rate for Tuberculosis (approximately 99.6%) is of paramount clinical importance. Since Tuberculosis is a highly contagious and severe respiratory disease, minimizing false negatives is critical for ensuring timely patient isolation and treatment. Ultimately, these results confirm that the WOA-driven structural search is capable of discovering highly reliable discriminatory patterns suitable for high-stakes medical environments.

It is noteworthy that misclassifications are minimal and mostly occur between “Normal” and “Viral Pneumonia”, which is clinically understandable due to subtle early-stage viral infiltrates that can mimic normal tissue. Importantly, the critical class of Tuberculosis is detected with near-perfect precision (only 1 miss in WOA), reinforcing the model’s utility for screening high-risk infectious diseases.

**Figure 24:**
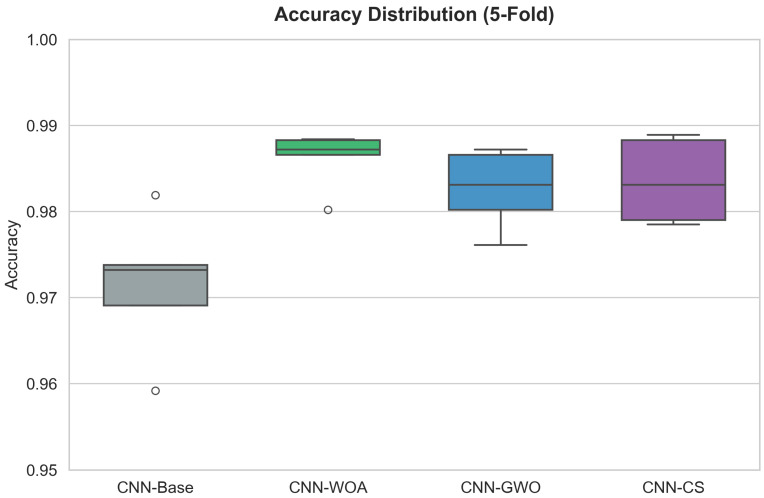
Accuracy distribution comparison for the 5-fold cross-validation scenario.

**Figure 25:**
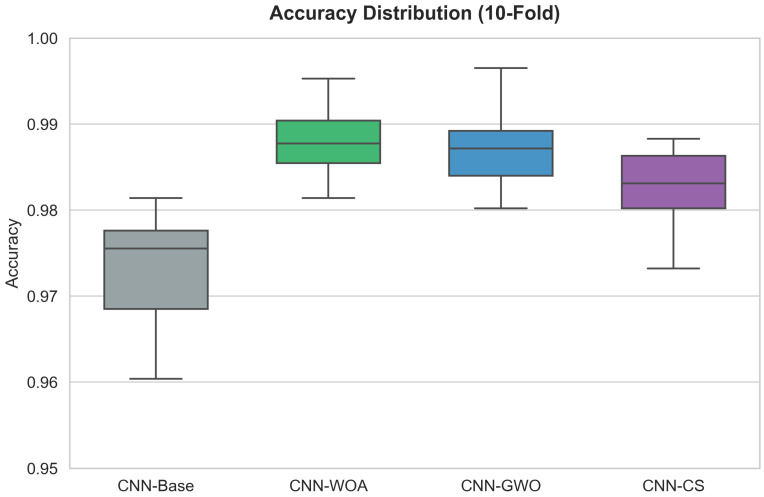
Accuracy distribution for 10-fold cross-validation showing the stability of CNN-WOA.

Consistent with these findings, the robustness of the proposed framework is further supported by the variability analysis across cross-validation folds. As visualized in the boxplots in Figure 24 and Figure 25, the optimized models exhibit a more compact interquartile range compared to the baseline, indicating greater stability and lower variability across folds. These figures provide a comparative view of model behavior under both 5-fold and 10-fold cross-validation protocols, highlighting the robustness of the optimized configurations across different data partitioning scenarios. This behavior confirms that the observed performance gains are consistent and not driven by a particular randomized data split.

The boxplots reveal critical insights regarding model reliability that average metrics alone cannot capture.

In the 5-Fold scenario (Figure 24), a clear hierarchy is established. The CNN-Base (grey box) sits noticeably lower on the accuracy scale, with a median below 0.975 and outlier points dropping near 0.96. In contrast, the metaheuristic-optimized models (WOA, GWO, CS) show distributions shifted upwards, with medians comfortably above 0.98. CNN-WOA (green box) stands out as particularly robust, with a very tight box indicating that its performance variance across folds is minimal.

The distinction becomes even more pronounced in the 10-Fold scenario (Figure 25). Here, the increased data fragmentation exposes the instability of the manual configuration. The CNN-Base box expands significantly, showing a wide interquartile range and whiskers extending down to 0.96, which implies that the model’s success is heavily dependent on the specific data partition. Conversely, CNN-WOA maintains its structural integrity; its distribution remains compact and elevated, with a median approaching 0.99. This behavior confirms that the hyperparameters found by the Whale Optimization Algorithm are not just “lucky” for a specific split but are genuinely robust, ensuring consistent high performance regardless of how the training data is subsetted. This low variance is a highly desirable attribute for trustworthy clinical deployment, minimizing the risk of performance degradation on unseen data.

### 6.3. Comparison with State-of-the-Art Lightweight Models

To rigorously contextualize the performance of the proposed metaheuristic approach, a comparative experiment was conducted against three modern, state-of-the-art lightweight architectures: ResNet-18, EfficientNet-B0, and MobileNetV3-Small. These models were evaluated under the exact same 10-fold cross-validation protocol and hardware constraints.

As shown in Table 10, the dynamically optimized CNN-WOA framework outperforms the standard lightweight models in overall Accuracy (98.83%). Notably, with 12.20 million parameters, the CNN-WOA maintains a structural complexity highly comparable to ResNet-18 (11.17 M) while achieving a decisively superior global accuracy (98.83% vs. 97.57%). While EfficientNet-B0 (4.01 M) demonstrates strong performance (97.80% accuracy), it falls short of the structural optimality found by the Whale Optimization Algorithm for this specific medical imaging task by a full percentage point. Furthermore, the MobileNetV3-Small architecture (1.52 M) exhibited severe instability during cross-validation, reflected in a massive standard deviation (±18.88%). This critical failure highlights a primary vulnerability of out-of-the-box pre-trained models when applied to specific clinical datasets without structural adaptation. Ultimately, this comparison mathematically validates that the WOA-driven architectural search is a robust method capable of discovering domain-specific topologies that clearly surpass established human-designed lightweight models.

### 6.4. Statistical Validation Results

To verify the significance of the results, the Wilcoxon signed-rank test was applied (α=0.05). Table 11 details the calculated *p*-values and the hypothesis decisions for both validation scenarios.

The analysis reveals the critical impact of sample size on statistical power in non-parametric tests:

- Impact of Validation Strategy: In the 5-fold scenario, despite the clear superiority of the optimized models in every fold, the test yielded a *p*-value of 0.0625. This value corresponds to the mathematical lower limit of the two-sided Wilcoxon test for N=5, preventing the rejection of the null hypothesis at the 95% confidence level purely due to sample size limitations.
- Confirmation of Improvement: By extending the validation to 10 folds (N=10), the statistical power increased sufficiently to reflect the true performance difference. As shown in Table 11, all metaheuristic models (WOA, GWO, CS) yielded *p*-values significantly lower than 0.05 (e.g., p=0.00195 for WOA) when compared to the baseline. Consequently, the null hypothesis ($H_{0}$) is rejected, statistically confirming that the proposed optimization provides a robust improvement over manual tuning.
- Model Comparison: The comparison between the top performers, CNN-WOA and CNN-GWO, resulted in *p*-values ≥ 0.05 in both scenarios. This indicates a statistical tie in diagnostic capability. However, given the results in Section 6.6, CNN-WOA remains the preferred choice due to its superior computational efficiency.

### 6.5. Hyperparameter Analysis

Table 12 details the optimal hyperparameter configurations discovered by each metaheuristic algorithm compared to the manually configured baseline.

A comprehensive examination of the results reveals a critical convergence pattern regarding the learning rate. Regardless of the validation protocol (5 or 10 folds), all metaheuristic-optimized models consistently selected learning rates in the range of $10^{-4}$ (0.0001–0.0004), which is an order of magnitude smaller than the baseline’s learning rate of 0.001. This strongly suggests that the manual configuration was too aggressive, likely causing the model to oscillate around the global minimum without settling.

In terms of the training duration, a clear correlation is observed between the learning rate and the number of epochs. The baseline model was limited to 20 epochs, which, combined with a high learning rate, likely resulted in suboptimal convergence. In contrast, the metaheuristic algorithms identified that a longer training phase was necessary to accommodate the smaller learning steps. Notably, CNN-WOA consistently selected the maximum number of epochs allowed in the search space (40 epochs) for both 5 and 10 folds. This extended training duration allowed the model to refine its weights more precisely without overfitting, as evidenced by the high validation accuracy.

The selection of batch size exhibited an interesting adaptive behavior depending on the validation strategy. In the 5-fold scenario, CNN-WOA and CNN-GWO favored smaller batch sizes (49 and 32, respectively). Smaller batches typically introduce more noise into the gradient estimation, which can act as a regularizer and help the model escape local minima. However, in the 10-fold scenario, CNN-WOA shifted towards a large batch size (256). This adjustment suggests that with more fragmented data, the algorithm prioritized gradient stability and computational speed, demonstrating the flexibility of the metaheuristic search to adapt hyperparameters to the specific constraints of the validation protocol.

Regarding the architectural topology, the impact of Batch Normalization (BN) is found to be consistent rather than marginal across all optimized configurations. As detailed in Table 12, the metaheuristic algorithms (WOA, GWO, and CS) universally selected the inclusion of BN for both 5-fold and 10-fold validation protocols.

This universal convergence suggests that BN is a fundamental structural component for the stability of the proposed custom CNN. Its primary role is to mitigate internal covariate shift, which facilitates the training of deeper architectures and ensures faster convergence by allowing the use of optimized learning rates. Unlike other hyperparameters that exhibited variability depending on the specific search logic of the metaheuristic, the constant selection of BN identifies it as a non-marginal factor for achieving high diagnostic performance in automated chest X-ray analysis.

Finally, concerning architectural topology, the results indicate that the baseline model was over-parameterized. The manual configuration employed 128 filters across 4 layers. In contrast, the best-performing model, CNN-WOA, reduced the number of filters to 64 in both scenarios. Additionally, the universal selection of Batch Normalization (True) across all optimized models confirms its critical role in mitigating internal covariate shift, facilitating the training of the deeper architectures favored by the algorithms in the 10-fold experiments.

### 6.6. Computational Efficiency and Model Complexity

In clinical deployment scenarios, particularly on edge devices or mobile systems, model storage footprint and inference speed are as critical as diagnostic accuracy. Table 13 compares the computational cost of the models in terms of Floating Point Operations (FLOPs), number of parameters, training time, and physical model size.

To provide a mathematical foundation for the empirical observations regarding computational efficiency, we analyze the complexity of the architectures from first principles. The computational cost (FLOPs) and the number of trainable parameters (P) of a standard convolutional layer *ℓ* are defined as follows:

(2) $$C_{flops}\left(\ell \right)\approx H_{\ell }\cdot W_{\ell }\cdot K_{\ell }^{2}\cdot C_{\ell ,in}\cdot C_{\ell ,out}$$

(3) $$P_{params}\left(\ell \right)=\left(K_{\ell }^{2}\cdot C_{\ell ,in}+1\right)\cdot C_{\ell ,out}$$

where H×W represents the feature map spatial dimensions, *K* is the kernel size, and $C_{in},C_{out}$ are the number of input and output channels, respectively.

Applying this formulation explains the architectural advantage of the CNN-WOA model. The manual baseline employed a fixed width of $C_{out}=128$ filters. In contrast, the metaheuristic optimization converged to a narrower configuration with $C_{out}=64$ filters. Since both FLOPs and Parameters depend linearly on the product of input and output channels ($C_{in}\cdot C_{out}$), halving the number of filters results in a drastic reduction in complexity, particularly in sequential layers where the input of layer L+1 depends on the output of layer *L*.

These theoretical deductions are confirmed by the empirical measurements presented in Table 13.

The results from the 5-fold validation highlight a significant advantage of the CNN-WOA model. It emerged as the most efficient architecture across almost all dimensions, requiring only 0.31 GFLOPs and occupying just 28.59 MB of storage. When compared to the baseline CNN (78.07 MB), the CNN-WOA achieves a reduction of approximately 63% in storage requirements and 74% in computational load. This finding confirms that the metaheuristic optimization successfully identified a compact architecture that generalizes better than the larger, manually configured baseline.

In the 10-fold evaluation, the behavior of the algorithms shifted. CNN-GWO converged to a very lightweight architecture (28.5 MB), similar to the best 5-fold model. However, as noted in the previous section, its diagnostic performance was slightly lower than that of CNN-WOA. Conversely, CNN-WOA adapted to the higher data fragmentation of 10 folds by selecting a slightly deeper architecture, resulting in a model size of 47.7 MB. Despite this increase, it remains 40% smaller than the baseline and provides the highest diagnostic accuracy of all tested configurations.

Regarding computational cost during training, CNN-CS exhibited a significant drawback in the 10-fold scenario, requiring nearly an hour (0:55:02) to complete the process, likely due to a less efficient convergence path. In contrast, CNN-WOA maintained a reasonable training time (approx. 25 min) even with the deeper architecture.

The superior performance of the CNN-WOA model across both 5-fold and 10-fold validation protocols is not an isolated occurrence, but a result of the algorithm’s unique search balance. Unlike Cuckoo Search, which relies heavily on random-walk exploration (Lévy flights) or GWO, which follows a rigid social hierarchy, WOA utilizes a bubble-net attacking mechanism that facilitates a more refined exploitation of the hyperparameter space.

This is evidenced by WOA’s ability to consistently identify a specific “sweet spot” in the architecture: a reduction to 64 filters combined with a maximum of 40 training epochs. While other metaheuristics fluctuated between suboptimal depths, WOA’s spiral update rule allowed it to settle into a lightweight configuration that maximizes diagnostic sensitivity without the overhead of the baseline. The fact that CNN-WOA maintained the lowest standard deviation (±0.30) confirms that its convergence is deterministic and robust, effectively dismissing the possibility that its success was influenced by a fortunate initialization or a specific data partition.

### 6.7. Visual Explainability with Grad-CAM++

**Figure 26:**
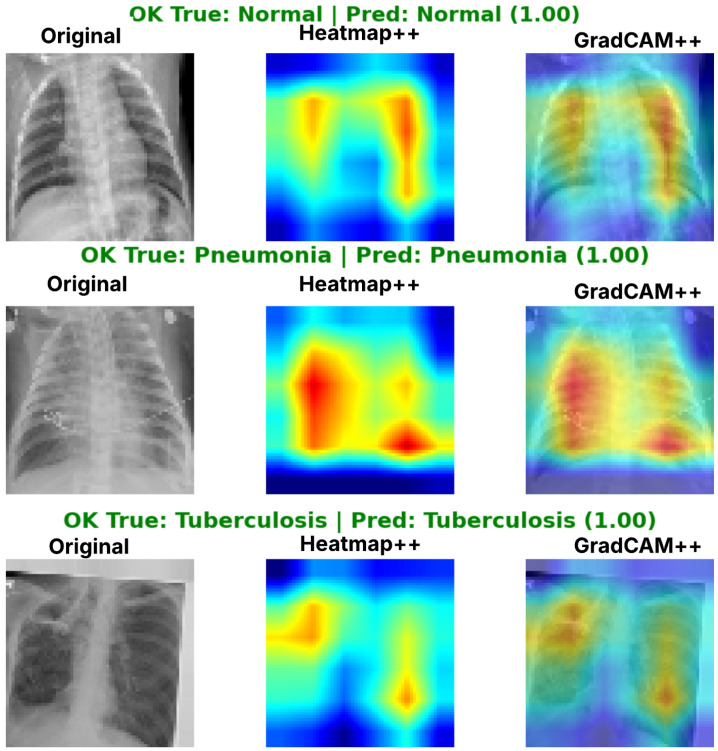
Grad-CAM++ visual explanations for the CNN-BASE model.

**Figure 27:**
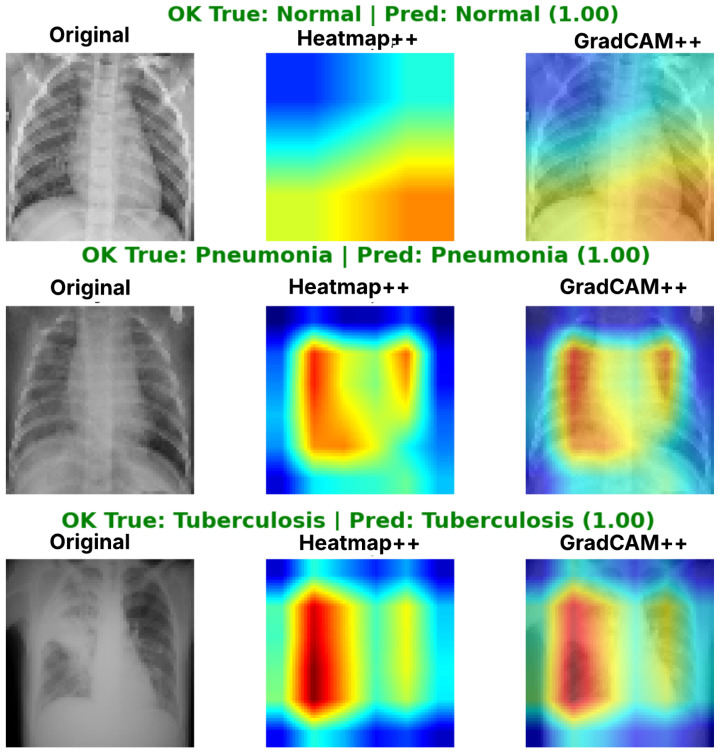
Grad-CAM++ visual explanations for the CNN-CS model.

**Figure 28:**
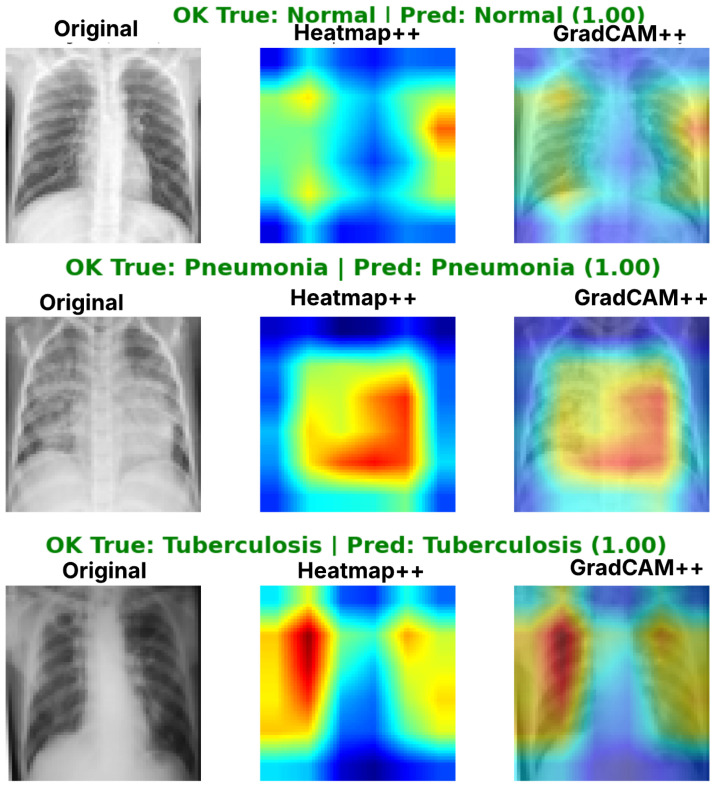
Grad-CAM++ visual explanations for the CNN-WOA model.

**Figure 29:**
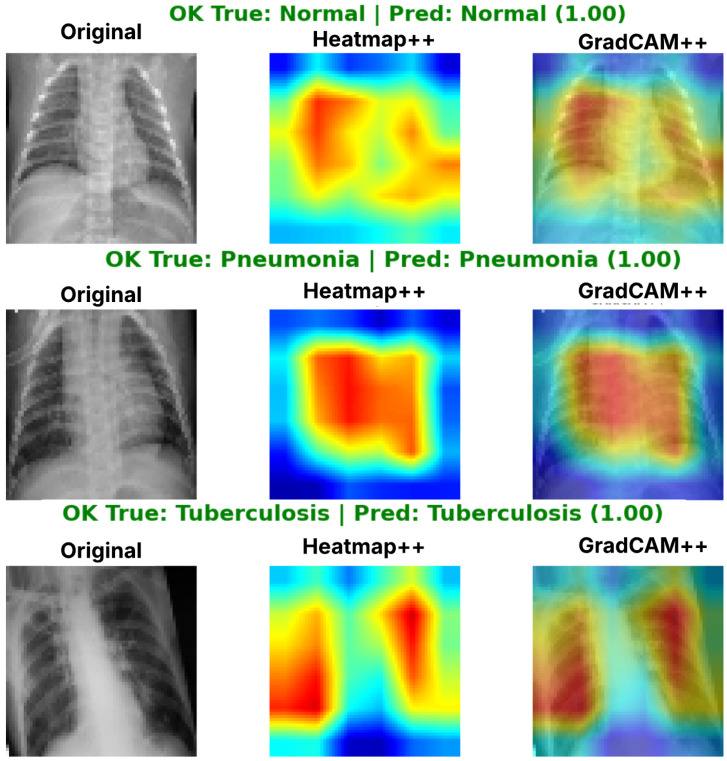
Grad-CAM++ visual explanations for the CNN-GWO model.

While quantitative metrics such as accuracy and sensitivity provide a measure of performance, they do not guarantee that the model is learning medically relevant features. To address the “black box” nature of Deep Learning and ensure clinical reliability, the decision-making process of the models was audited using Grad-CAM++. For all Grad-CAM++ and Heatmap++ visualizations presented in the following figures, warmer colors (red/yellow) indicate regions with higher activation and stronger contribution to the model prediction, whereas cooler colors (blue) correspond to lower activation areas. Figure 26, Figure 27, Figure 28 and Figure 29 illustrate the activation maps across the three diagnostic classes for the CNN-Base (Figure 26), CNN-CS (Figure 27), CNN-WOA (Figure 28) and CNN-GWO (Figure 29) models.

This comparative visual analysis corroborates that the metaheuristic optimization process successfully guided the networks to focus on the lung parenchyma rather than confounding artifacts, with notable differences in precision depending on the specific search algorithm employed.

- CNN-Base: The unoptimized baseline model serves as a critical counter-example, highlighting the necessity of metaheuristic tuning. The Grad-CAM++ overlays for the baseline model demonstrate a severe tendency to focus on confounding artifacts. In the Normal case, the network incorrectly places its highest attention on the upper right clavicle and the extreme borders of the rib cage. In the Tuberculosis case, alongside a relevant focal point in the apex, it presents random attention hotspots on the extreme lower left edge, outside the primary parenchymal zones.
- CNN-CS: The Cuckoo Search optimized model successfully localizes bilateral anomalies in Tuberculosis; however, its attention boundaries are more diffuse and exhibit a “blocky” pattern. The Lévy flight exploration allows CS to avoid local minima effectively, but the resulting convolutional filters appear to capture broader textures rather than sharp pathological edges. In Viral Pneumonia cases, although the focus is on the lower lung fields, the activation lacks the anatomical contouring seen in the WOA model, explaining its marginally lower sensitivity scores.
- CNN-WOA: As the top-performing architecture, CNN-WOA exhibits the most precise and clinically coherent attention mechanism. For Tuberculosis, the attention is intensely concentrated on the specific vertical lobar regions (bilateral), accurately tracing the lung fields where consolidations naturally occur, while remarkably avoiding the cardiac silhouette and central spine. In the case of Viral Pneumonia, the heatmap reveals a strong, diffuse activation pattern covering the central and lower lung fields, which is highly consistent with the manifestation of diffuse interstitial infiltrates. Normal cases show a properly dispersed attention without any severe pathological hotspots, indicating a comprehensive scan of the thoracic cavity.
- CNN-GWO: While the Grey Wolf Optimizer identifies the pathological zones, its activation maps demonstrate a tendency toward over-concentration. In Tuberculosis detection, the model focuses correctly on the affected lobes but exhibits a more centralized and dense hotspot compared to WOA. Notably, in Viral Pneumonia, GWO produces a massive activation region that slightly overlaps with the mediastinum. This suggests that while GWO finds the correct global region, it lacks the fine-tuned precision of the spiral search mechanism found in WOA, leading to slightly less localized features.

In conclusion, the comparative visual evidence confirms that the superior quantitative performance of the metaheuristic models, particularly CNN-WOA, is directly linked to genuine, artifact-free radiological feature learning, thereby supporting its reliability for real-world clinical deployment.

### 6.8. Extended Multi-Class Evaluation (6 Class Scenario)

To address the clinical reality of a busy triage environment where multiple pulmonary pathologies coexist (e.g., COVID-19, Bacterial Pneumonia, and Emphysema), an extended evaluation was conducted utilizing the full ChestX6 dataset encompassing all six classes. Maintaining methodological consistency with the core experiments of this study, the optimized CNN-GWO architecture was evaluated under both 5-fold and 10-fold crossvalidation protocols to rigorously assess its scalability and diagnostic reliability.

Table 14 details the fold-by-fold performance for the 5-fold scenario, while Table 15 presents the results under the more fragmented 10-fold stress test.

As observed in the tables, the model demonstrates a robust diagnostic capacity even when the classification complexity is doubled. In the 5-fold scenario, the model achieves an average accuracy of 89.26% and a high specificity of 0.9763. Similarly, the 10-fold test submit a resilient average accuracy of 88.60% and a specificity of 0.9733. While there is an expected natural decrease in overall accuracy compared to the highly specialized 3 class baseline, the model successfully maintains high specificity and an AUC consistently above 0.98. This confirms that the metaheuristic optimized architecture does not suffer from catastrophic confusion when introduced to similar radiological subtypes, reliably minimizing false positives across a diverse set of conditions, which is paramount for patient safety in real-world clinical deployments.

## 7. Conclusions

This study presented a hybrid framework that integrates Convolutional Neural Networks with metaheuristic optimization algorithms (WOA, GWO, and CS) to automate the diagnosis of Viral Pneumonia and Tuberculosis. The experimental results, validated through a rigorous stratified 5-fold and 10-fold cross-validation protocol, confirm that the proposed automated optimization significantly outperforms traditional manual hyperparameter tuning. Validating this improvement, the Wilcoxon signed-rank test demonstrated a statistically significant difference (p<0.05) in the 10-fold scenario, leading to the rejection of the null hypothesis. Specifically, the Whale Optimization Algorithm (WOA) emerged as the most robust strategy, achieving the highest diagnostic accuracy and specificity across all test scenarios, proving its capability to effectively navigate the non-convex search space of deep learning models.

Beyond diagnostic precision, a critical contribution of this work is the substantial improvement in computational efficiency. The optimization process successfully identified a lightweight architecture that reduced the storage requirement by approximately 63% and the computational load (FLOPs) by 74% compared to the baseline manual model. This demonstrates that high-performance medical models do not necessarily require heavy architectures, making the CNN-WOA model a viable candidate for deployment in resource-constrained environments, such as mobile health units or edge devices.

Finally, the integration of Explainable AI (XAI) through Grad-CAM++ provided necessary transparency to the “black box” nature of the model. The visual evidence confirmed that the optimized network learns to focus on clinically relevant structures—such as the lung parenchyma and specific lobar consolidations—while correctly ignoring confounding artifacts like bones or medical devices. This alignment between the model’s attention and radiological findings is essential for building trust among medical practitioners and facilitating the adoption of AI as a reliable second opinion in clinical workflows.

Based on the findings of this study, several key research directions have been identified to enhance the avenues to broaden the clinical applicability and technical robustness of the proposed framework:

- Expanded Pathological Scope. Incorporate a wider spectrum of pulmonary conditions, such as COVID-19, pneumothorax, and lung malignancies, to evaluate the scalability of the metaheuristic search in higher-dimensional multi-class scenarios.
- Architectural Hybridization. Investigate the optimization of hybrid architectures that combine Convolutional Neural Networks with Vision Transformers (ViT), aiming to synergize the local feature extraction of convolutions with the global attention mechanisms of Transformers.
- Multi-objective Optimization. Implement multi-objective algorithms, such as NSGA-II, to explicitly search for Pareto-optimal solutions that balance diagnostic sensitivity against model complexity (FLOPs), automating the design of efficient models for edge deployment.
- Prospective Clinical Validation. Transition from retrospective analysis to human-in-the-loop evaluations, conducting studies to measure the tangible impact of the model’s assistance and Grad-CAM++ visualizations on the diagnostic speed and accuracy of radiologists in clinical settings. While current explainability analyses based on Grad-CAM++ provide strong algorithmic transparency, formal validation protocols involving expert radiologists are imperative to confirm clinical efficacy and definitively bridge the gap to real-world deployment.
- Transition to Semantic Segmentation. Advance from weak localization via Grad-CAM++ to fully supervised semantic segmentation using specialized medical imaging architectures (e.g., U-Net or TransUNet), aiming to precisely delineate the boundaries of pulmonary infiltrates and cavitations for quantitative severity assessment.

Addressing these research directions will enable the proposed hybrid framework to evolve into a robust, efficient, and clinically deployable decision-support system for the automated screening of infectious pulmonary diseases, effectively bridging the gap between algorithmic innovation and the seamless integration of trustworthy AI within real-world radiological workflows.

## Figures and Tables

**Table 1:** CNN Fixed Architecture Parameters.

Component	Parameter Description
Input Image	Grayscale X-ray image with 1 channel
Convolutional Block	3 × 3 filters with padding = 1 and stride = 1, repeated with increasing filters.
Activation Function	ReLU function introduces non-linearity after each convolution.
Pooling Operation	MaxPooling 2 × 2 reduces spatial dimensions.
Fully Connected Layer 1	Dense layer with 1024 units for feature aggregation.
Dropout	Dropout rate of 0.5 applied after FC1 to reduce overfitting.
Fully Connected Layer 2	Dense layer with 3 output units for multi-class classification.
Output	Log-Softmax generates log-probabilities over the output classes.

**Table 2:** Evaluation metrics and their definitions.

Metric	Description	Equation
Recall (Sensitivity)	Proportion of actual positives correctly identified [36]	$\frac{TP}{TP+FN}$
Specificity	Proportion of actual negatives correctly classified [14]	$\frac{TN}{TN+FP}$
F1-Score	Harmonic mean of precision and recall [28]	$2\cdot \frac{\mathrm{Precision}\cdot \mathrm{Recall}}{\mathrm{Precision}+\mathrm{Recall}}$
AUC	Area under the ROC curve [4]	$\int_{0}^{1}TPR\left(FPR\right)\;dFPR$
Precision	Proportion of predicted positives correctly identified [52]	$\frac{TP}{TP+FP}$

**Table 3:** ChestX6: Multi-Class X-ray Dataset.

Class	Train	Val	Test	Total
Normal	2671	300	300	3271
Pneumonia-Bacterial	2400	300	300	3000
Pneumonia-Viral	2413	300	300	3013
COVID-19	2417	300	300	3017
Tuberculosis	2600	298	287	3185
Emphysema	2050	250	250	2550
Total	14,551	1748	1737	18,036

**Table 4:** Distribution of the dataset.

Class	Train	Val	Test	Total
Normal	2671	300	300	3271
Pneumonia-Viral	2413	300	300	3013
Tuberculosis	2600	298	287	3185
Total	7684	898	887	9469

**Table 5:** Hyperparameters subject to optimization.

Component	Parameter	Search Space
CNN Architecture	Number of convolutional layers	{3, 4, 5}
	Filters per layer	{64, 128, 256}
	Use of Batch Normalization (BN)	{True, False}
Training	Learning Rate	(0.0001, 0.1)
	Batch Size	{64, 128, 256}
	Epochs	(20, 40)

**Table 6:** Optimization schemes for metaheuristics.

MH	Folds	Population	Iterations
GWO	5	10	10
CS	5	10	10
WOA	5	10	10
GWO	10	10	10
CS	10	10	10
WOA	10	10	10

**Table 7:** Comparative performance results (Mean ± STD) on 5 and 10 folds.

Model	Folds	Accuracy	Recall	Specificity	F1-Score	AUC	Presicion
CNN-GWO	5	0.9826±0.41	0.9834±0.0046	0.9834±0.0046	0.9675±0.0091	0.9976±0.0006	0.9676±0.021
CNN-CS	5	0.9836±0.44	0.9688±0.0125	0.9841±0.0064	0.9688±0.0127	0.9973±0.0004	0.9688±0.028
CNN-WOA	5	0.9861±0.30	0.9808±0.0016	0.9902±0.0008	0.9809±0.0016	0.9981±0.0003	0.9810±0.004
CNN-Base	5	0.9715±0.74	0.9445±0.0248	0.9717±0.0127	0.9440±0.0258	0.9959±0.0010	0.9435±0.058
CNN-GWO	10	0.9871±0.47	0.9729±0.0148	0.9862±0.0076	0.9729±0.0150	0.9982±0.0004	0.9729±0.034
CNN-CS	10	0.9825±0.44	0.9736±0.0047	0.9865±0.0024	0.9737±0.0047	0.9975±0.0005	0.9738±0.011
CNN-WOA	10	0.9883±0.41	0.9818±0.0016	0.9907±0.0008	0.9819±0.0016	0.9982±0.0004	0.9820±0.003
CNN-Base	10	0.9724±0.69	0.9453±0.0381	0.9722±0.0194	0.9445±0.0409	0.9956±0.0029	0.9437±0.092

**Table 8:** Comparative performance results (Mean ± STD [95% CI]) on 5 and 10 folds.

Model	Folds	Accuracy	Recall	Specificity	F1-Score	AUC	Precision
CNN-GWO	5	0.9826±0.0041	0.9834±0.0046	0.9834±0.0046	0.9675±0.0091	0.9976±0.0006	0.9676±0.0210
		[0.978, 0.988]	[0.978, 0.989]	[0.978, 0.989]	[0.956, 0.979]	[0.997, 0.998]	[0.941, 0.994]
CNN-CS	5	0.9836±0.0044	0.9688±0.0125	0.9841±0.0064	0.9688±0.0127	0.9973±0.0004	0.9688±0.0280
		[0.978, 0.989]	[0.953, 0.984]	[0.976, 0.992]	[0.953, 0.985]	[0.997, 0.998]	[0.934, 1.000]
CNN-WOA	5	0.9861±0.0030	0.9808±0.0016	0.9902±0.0008	0.9809±0.0016	0.9981±0.0003	0.9810±0.0040
		[0.982, 0.990]	[0.979, 0.983]	[0.989, 0.991]	[0.979, 0.983]	[0.998, 0.998]	[0.976, 0.986]
CNN-Base	5	0.9715±0.0074	0.9445±0.0248	0.9717±0.0127	0.9440±0.0258	0.9959±0.0010	0.9435±0.0580
		[0.962, 0.981]	[0.914, 0.975]	[0.956, 0.987]	[0.912, 0.976]	[0.995, 0.997]	[0.871, 1.000]
CNN-GWO	10	0.9871±0.0047	0.9729±0.0148	0.9862±0.0076	0.9729±0.0150	0.9982±0.0004	0.9729±0.0340
		[0.984, 0.991]	[0.962, 0.984]	[0.981, 0.992]	[0.962, 0.984]	[0.998, 0.998]	[0.949, 0.997]
CNN-CS	10	0.9825±0.0044	0.9736±0.0047	0.9865±0.0024	0.9737±0.0047	0.9975±0.0005	0.9738±0.0110
		[0.979, 0.986]	[0.970, 0.977]	[0.985, 0.988]	[0.970, 0.977]	[0.997, 0.998]	[0.966, 0.982]
CNN-WOA	10	0.9883±0.0041	0.9818±0.0016	0.9907±0.0008	0.9819±0.0016	0.9982±0.0004	0.9820±0.0030
		[0.985, 0.991]	[0.981, 0.983]	[0.990, 0.991]	[0.981, 0.983]	[0.998, 0.998]	[0.980, 0.984]
CNN-Base	10	0.9724±0.0069	0.9453±0.0381	0.9722±0.0194	0.9445±0.0409	0.9956±0.0029	0.9437±0.0920
		[0.967, 0.977]	[0.918, 0.973]	[0.958, 0.986]	[0.915, 0.974]	[0.993, 0.998]	[0.878, 1.000]

**Table 9:** Ablation analysis: comparative contribution of metaheuristic optimization to model performance (5-fold validation).

Model	Optimization Type	Accuracy	Recall	F1-Score	AUC
CNN-Base	None	0.9715	0.9445	0.9440	0.9959
CNN-CS	Metaheuristic	0.9836	0.9688	0.9688	0.9973
CNN-GWO	Metaheuristic	0.9826	0.9834	0.9675	0.9976
CNN-WOA	Metaheuristic	0.9861	0.9808	0.9809	0.9981

**Table 10:** Comparison of the proposed CNN-WOA against modern lightweight pre-trained models (10 Folds).

Model	Accuracy	Precision	Recall	Specificity	F1-Score	AUC
CNN-WOA	0.9883 ± 0.0041	0.9820 ± 0.0030	0.9818 ± 0.0016	0.9907 ± 0.0008	0.9819 ± 0.0016	0.9982 ± 0.0004
EfficientNet-B0	0.9780 ± 0.0017	0.9883 ± 0.0017	0.9882 ± 0.0017	0.9940 ± 0.0008	0.9882 ± 0.0017	0.9983 ± 0.0010
ResNet-18	0.9757 ± 0.0047	0.9860 ± 0.0046	0.9859 ± 0.0047	0.9928 ± 0.0024	0.9859 ± 0.0046	0.9987 ± 0.0004
MobileNetV3-Small	0.8238 ± 0.1888	0.7979 ± 0.2316	0.8251 ± 0.1885	0.9114 ± 0.0950	0.7935 ± 0.2344	0.9371 ± 0.0916

**Table 11:** Statistical validation results using Wilcoxon signed-rank test (α=0.05).

Comparison	5-Fold Validation		10-Fold Validation	
	p-Value	Decision ($H_{0}$)	p-Value	Decision ($H_{0}$)
CNN-WOA vs. CNN-Base	0.0625	Fail to Reject	0.00195	Reject
CNN-GWO vs. CNN-Base	0.0625	Fail to Reject	0.00195	Reject
CNN-CS vs. CNN-Base	0.0625	Fail to Reject	0.00586	Reject
CNN-WOA vs. CNN-GWO	0.1250	Fail to Reject	0.24219	Fail to Reject

**Table 12:** Hyperparameter settings for the evaluated CNN models.

Model	Folds	Num_Layers	Num_Filters	Batch_Norm	Epochs	Batch_Size	lr
CNN-GWO	5	3	122	True	31	32	0.0001
CNN-CS	5	5	64	True	24	128	0.0003
CNN-WOA	5	4	64	True	40	64	0.0001
CNN-Base	5	4	128	True	20	128	0.001
CNN-GWO	10	4	64	True	25	256	0.0004
CNN-CS	10	4	128	False	36	128	0.0001
CNN-WOA	10	5	64	True	40	256	0.0001
CNN-Base	10	4	128	True	20	128	0.001

**Table 13:** Model performance metrics regarding efficiency.

Model	Folds	Flops	Params	Time	Model Size
CNN-GWO	5	0.84 GFLOPs	26.88 M	0:20:58	102.5 MB
CNN-CS	5	0.34 GFLOPs	12.20 M	0:13:51	46.56 MB
CNN-WOA	5	0.31 GFLOPs	7.48 M	0:15:57	28.59 MB
CNN-Base	5	1.21 GFLOPs	20.46 M	0:15:03	78.07 MB
CNN-GWO	10	0.31 GFLOPs	7.48 M	0:28:46	28.5 MB
CNN-CS	10	1.20 GFLOPs	20.46 M	0:55:02	79.9 MB
CNN-WOA	10	0.34 GFLOPs	12.20 M	0:24:54	47.7 MB
CNN-Base	10	1.21 GFLOPs	20.46 M	0:22:18	78.07 MB

**Table 14:** Detailed fold-by-fold performance of the proposed model on the extended 6-class dataset (5-fold crossvalidation).

Fold	Test Accuracy (%)	F1-Score	AUC
Fold 1	89.52	0.8963	0.9878
Fold 2	86.47	0.8670	0.9856
Fold 3	89.87	0.9000	0.9887
Fold 4	89.93	0.9009	0.9896
Fold 5	85.03	0.8489	0.9844
Global Metrics	Accuracy: 89.26% ± 2.27 Recall: 0.8840		
(Average ± SD)	Specificity: 0.9763 F1-Score: 0.8826 ± 0.0234 AUC: 0.9872 ± 0.0022		

**Table 15:** Detailed fold-by-fold performance of the proposed model on the extended 6-class dataset (10-fold crossvalidation).

Fold	Test Accuracy (%)	F1-Score	AUC
Fold 1	90.85	0.9095	0.9919
Fold 2	83.42	0.8288	0.9822
Fold 3	87.74	0.8757	0.9889
Fold 4	78.30	0.7876	0.9751
Fold 5	87.10	0.8720	0.9876
Fold 6	90.27	0.9033	0.9909
Fold 7	84.34	0.8404	0.9851
Fold 8	91.13	0.9129	0.9916
Fold 9	89.06	0.8904	0.9912
Fold 10	84.74	0.8442	0.9844
Global Metrics	Accuracy: 88.60% ± 4.04 Recall: 0.8673		
(Average ± SD)	Specificity: 0.9733 F1-Score: 0.8665 ± 0.0406 AUC: 0.9869 ± 0.0053		

## Data Availability

The datasets analyzed in this study are publicly available. The ChestX6 dataset can be accessed at https://www.kaggle.com/datasets/mohamedasak/chest-x-ray-6-classes-dataset (accessed on 15 August 2025). The source code used for model training, metaheuristic optimization, and evaluation is publicly available on GitHub at https://github.com/lukipuki0/Pneumonia_tuberculosis (accessed on 10 May 2026).

## Abbreviations

The following abbreviations are used in this manuscript:

AI	Artificial Intelligence
AUC	Area Under the ROC Curve
BN	Batch Normalization
CNN	Convolutional Neural Network
CS	Cuckoo Search
CV	Cross-Validation
CXR	Chest X-ray
DL	Deep Learning
F1	F1-Score
FP	False Positive
FN	False Negative
FPR	False Positive Rate
GWO	Grey Wolf Optimizer
Grad-CAM++	Gradient-weighted Class Activation Mapping++
HPO	Hyperparameter Optimization
*k*-fold	*k*-fold Cross-Validation
MCC	Matthews Correlation Coefficient
ReLU	Rectified Linear Unit
ROC	Receiver Operating Characteristic
TB	Tuberculosis
TL	Transfer Learning
TP	True Positive
TN	True Negative
TPR	True Positive Rate
WOA	Whale Optimization Algorithm
XAI	Explainable Artificial Intelligence
